# Supplementary material for: Polyketide synthase gene clusters in the ericoid mycorrhizal fungus Oidiodendron maius: genome-wide prediction, expression profiling, and functional characterization of knockout mutants
Source: Front Plant Sci. 2026 Sep 16;17:1924430. doi: 10.3389/fpls.2026.1924430 (PMC13623903; doi:10.3389/fpls.2026.1924430)
Supplement: Supplementary file 1 [file SupplementaryFile1.zip › Supplementary Materials/Supplementary Folder 1.zip!/Oidma1_AssemblyScaffolds_Repeatmasked/knownclusterblast/cluster35/jgi.p_Oidma1_131360_mibig_hits.html]

| MIBiG Protein | Description | MIBiG Cluster | MiBiG Product | % ID | % Coverage | BLAST Score | E-value |
| --- | --- | --- | --- | --- | --- | --- | --- |
| EGD99348.1 | polyketide\_synthase | BGC0001144 | Neosartoricin\_biosynthetic\_gene\_cluster | 71 | 100.832870627 | 2528 | 0.0 |
| CBF70387.1 | polyketide\_synthase,\_putative\_(JCVI) | BGC0000684 | Asperthecin\_biosynthetic\_gene\_cluster | 68 | 100.499722376 | 2419 | 0.0 |
| AEN83889.1 | AdaA | BGC0000156 | TAN-1612\_biosynthetic\_gene\_cluster | 67 | 100.943920044 | 2393 | 0.0 |
| ADI24926.1 | VrtA | BGC0000168 | Viridicatumtoxin\_biosynthetic\_gene\_cluster | 63 | 101.665741255 | 2313 | 0.0 |
| CBF90097.1 | hypothetical\_protein\_similar\_to\_polyketide\_synthase\_(Broad) | BGC0001271 | Emericellin\_biosynthetic\_gene\_cluster | 50 | 101.332593004 | 1698 | 0.0 |
| EAL89339.1 | polyketide\_synthase,\_putative | BGC0001403 | Trypacidin\_biosynthetic\_gene\_cluster | 49 | 100.111049417 | 1661 | 0.0 |
| AGO59040.1 | PtaA | BGC0000121 | Pestheic\_acid\_biosynthetic\_gene\_cluster | 48 | 95.6690727374 | 1586 | 0.0 |
| CBF79143.1 | polyketide\_synthase,\_putative\_(JCVI) | BGC0000013 | Alternariol\_biosynthetic\_gene\_cluster | 47 | 104.108828429 | 1534 | 0.0 |
| EAL84397.1 | polyketide\_synthase,\_putative | BGC0001118 | Endocrocin\_biosynthetic\_gene\_cluster | 45 | 100.166574125 | 1527 | 0.0 |
| AKN45693.1 | polyketide\_synthase | BGC0001284 | Alternariol\_biosynthetic\_gene\_cluster | 42 | 100.277623542 | 1347 | 0.0 |
| EED53479.1 | polyketide\_synthase,\_putative | BGC0001304 | Aflavarin\_biosynthetic\_gene\_cluster | 42 | 99.7223764575 | 1320 | 0.0 |
| ADI24953.1 | GsfA | BGC0000070 | Griseofulvin\_biosynthetic\_gene\_cluster | 40 | 101.277068295 | 1279 | 0.0 |
| BAD22832.1 | polyketide\_synthase | BGC0001265 | Melanin\_biosynthetic\_gene\_cluster | 41 | 92.2265408107 | 1228 | 0.0 |
| ABU63483.1 | polyketide\_synthase | BGC0001259 | Elsinochrome\_A\_biosynthetic\_gene\_cluster | 39 | 99.444752915 | 1220 | 0.0 |
| AAN59953.1 | polyketide\_synthase\_1 | BGC0001258 | 1,8-dihydroxynaphthalene\_biosynthetic\_gene\_cluster | 41 | 91.6157690172 | 1209 | 0.0 |
| AAD38786.1 | polyketide\_synthase | BGC0001257 | 1,3,6,8-tetrahydroxynaphthalene\_biosynthetic\_gene\_cluster | 39 | 98.8895058301 | 1203 | 0.0 |
| EAU38791.1 | hypothetical\_protein | BGC0000161 | Terrein\_biosynthetic\_gene\_cluster | 39 | 91.6157690172 | 1121 | 0.0 |
| CBF74114.1 | Conidial\_yellow\_pigment\_biosynthesis\_polyketide\_synthase\_(PKS)(EC\_2.3.1.-)\_[Source:UniProtKB/Swiss-Prot;Acc:Q03149] | BGC0000107 | Naphthopyrone\_biosynthetic\_gene\_cluster | 38 | 93.4480843976 | 1085 | 0.0 |
| ADM79459.1 | PKS16\_protein | BGC0001266 | Grayanic\_acid\_biosynthetic\_gene\_cluster | 38 | 94.503053859 | 1065 | 8.2e-311 |
| CCT67991.1 | bikaverin\_cluster-polyketide\_synthase | BGC0000030 | Bikaverin\_biosynthetic\_gene\_cluster | 36 | 98.7229317046 | 1037 | 1.4e-302 |
| CCE67070.1 | polyketide\_synthase | BGC0001242 | Fusarubin\_biosynthetic\_gene\_cluster | 38 | 91.3936701832 | 1010 | 1.8e-294 |
| CCH26295.1 | putative\_polyketide\_synthase | BGC0001158\_c6 | Bikaverin\_biosynthetic\_gene\_cluster | 35 | 98.7784564131 | 988 | 9.8e-288 |
| AAT69682.1 | polyketide\_synthase | BGC0001247 | Cercosporin\_biosynthetic\_gene\_cluster | 35 | 101.610216546 | 946 | 4.3e-275 |
| AAS90093.1 | PksA | BGC0000006 | Aflatoxin\_biosynthetic\_gene\_cluster | 37 | 76.124375347 | 848 | 1.2e-245 |
| BAE71314.1 | polyketide\_synthase | BGC0000004 | Aflatoxin\_biosynthetic\_gene\_cluster | 37 | 76.124375347 | 842 | 1.1e-243 |
| AAS90022.1 | PksA | BGC0000008 | Aflatoxin\_biosynthetic\_gene\_cluster | 37 | 76.124375347 | 841 | 1.5e-243 |
| AAS89999.1 | PksA | BGC0000007 | Aflatoxin\_biosynthetic\_gene\_cluster | 37 | 76.2909494725 | 840 | 3.3e-243 |
| AAS90047.1 | PksA | BGC0000009 | Aflatoxin\_biosynthetic\_gene\_cluster | 37 | 76.2909494725 | 832 | 9e-241 |
| AAZ95017.1 | polyketide\_synthase | BGC0000048 | Dothistromin\_biosynthetic\_gene\_cluster | 38 | 72.2931704609 | 830 | 3.4e-240 |
| ACH72912.1 | AflC | BGC0000011 | Aflatoxin/sterigmatocystin\_biosynthetic\_gene\_cluster | 36 | 81.1215991116 | 822 | 9.3e-238 |
| AAC49191.1 | putative\_polyketide\_synthase | BGC0000152 | Sterigmatocystin\_biosynthetic\_gene\_cluster | 35 | 84.4530816213 | 812 | 1.3e-234 |
| AAS92537.1 | PKS1 | BGC0001044 | Sirodesmin\_biosynthetic\_gene\_cluster | 34 | 82.398667407 | 769 | 1.2e-221 |
| EAA59563.1 | hypothetical\_protein | BGC0000057 | F9775\_biosynthetic\_gene\_cluster | 32 | 99.444752915 | 748 | 2.2e-215 |
| AGC95321.1 | CurS2 | BGC0000045 | Dehydrocurvularin\_biosynthetic\_gene\_cluster | 33 | 91.1715713493 | 745 | 1.1e-214 |
| ACD39762.1 | non-reducing\_polyketide\_synthase | BGC0000077 | Hypothemycin\_biosynthetic\_gene\_cluster | 31 | 100.0 | 743 | 7.2e-214 |
| ACD39753.1 | non-reducing\_polyketide\_synthase | BGC0000076 | Hypothemycin\_biosynthetic\_gene\_cluster | 31 | 100.0 | 742 | 9.4e-214 |
| AHV78247.1 | LasS2 | BGC0001245 | Lasiodiplodin\_biosynthetic\_gene\_cluster | 30 | 100.444197668 | 728 | 2.4e-209 |
| ABB90282.1 | polyketide\_synthase | BGC0001057 | Zearalenone\_biosynthetic\_gene\_cluster | 30 | 90.1721265963 | 715 | 2.1e-205 |
| ACD39770.1 | non-reducing\_polyketide\_synthase | BGC0000134 | Radicicol\_biosynthetic\_gene\_cluster | 31 | 91.1160466408 | 713 | 6.1e-205 |
| AHV78253.1 | ResS2 | BGC0001246 | Trans-resorcylide\_biosynthetic\_gene\_cluster | 32 | 82.6762909495 | 698 | 1.6e-200 |
| AAK57187.1 | MxaC | BGC0001022 | Myxalamid\_biosynthetic\_gene\_cluster | 36 | 49.9167129373 | 527 | 8.1e-149 |
| XP\_011392701.1 | hypothetical\_protein | BGC0001281 | Ustilagic\_acid\_biosynthetic\_gene\_cluster | 29 | 76.790671849 | 503 | 1.3e-141 |
| AGC45620.1 | polyketide\_synthase | BGC0001394 | Phenalamide\_biosynthetic\_gene\_cluster | 35 | 49.7501388118 | 501 | 3.6e-141 |
| ABM21570.1 | CrpB | BGC0000975 | Cryptophycin\_biosynthetic\_gene\_cluster | 33 | 55.4691837868 | 489 | 1.4e-137 |
| CAQ18829.1 | polyketide\_synthase | BGC0000954 | Ajudazol\_biosynthetic\_gene\_cluster | 35 | 50.7495835647 | 487 | 5.4e-137 |
| CAQ18834.1 | polyketide\_synthase | BGC0000954 | Ajudazol\_biosynthetic\_gene\_cluster | 36 | 49.7501388118 | 485 | 2.7e-136 |
| AHB82064.1 | polyketide\_synthase | BGC0001231 | Microsclerodermins\_biosynthetic\_gene\_cluster | 35 | 50.6940588562 | 485 | 2.7e-136 |
| CAD19087.1 | StiC\_protein | BGC0000153 | Stigmatellin\_biosynthetic\_gene\_cluster | 35 | 53.8034425319 | 483 | 1.3e-135 |
| CAQ18835.1 | polyketide\_synthase | BGC0000954 | Ajudazol\_biosynthetic\_gene\_cluster | 35 | 52.1932259856 | 482 | 2.3e-135 |
| AAT70103.1 | CurH | BGC0001165 | Curacin\_biosynthetic\_gene\_cluster | 34 | 50.4719600222 | 478 | 4.3e-134 |
| AEE88282.1 | CurH | BGC0000976 | Curacin\_biosynthetic\_gene\_cluster | 34 | 50.4719600222 | 478 | 4.3e-134 |
| AIW82282.1 | PuwE | BGC0001125 | Puwainaphycins\_biosynthetic\_gene\_cluster | 34 | 50.2498611882 | 477 | 7.3e-134 |
| AAY42396.1 | HctD | BGC0001000 | Hectochlorin\_biosynthetic\_gene\_cluster | 35 | 50.0277623542 | 475 | 2.8e-133 |
| CAQ34920.1 | polyketide\_synthase | BGC0000986 | Dkxanthene\_biosynthetic\_gene\_cluster | 35 | 52.5263742365 | 474 | 6.2e-133 |
| AAT70105.1 | CurJ | BGC0001165 | Curacin\_biosynthetic\_gene\_cluster | 34 | 51.3048306496 | 473 | 1.4e-132 |
| AEE88280.1 | CurJ | BGC0000976 | Curacin\_biosynthetic\_gene\_cluster | 34 | 51.3048306496 | 473 | 1.4e-132 |
| AAF19814.1 | MtaF | BGC0001024 | Myxothiazol\_biosynthetic\_gene\_cluster | 33 | 51.3603553581 | 472 | 2.4e-132 |
| AEU11006.1 | NpnB | BGC0001029 | Nostophycin\_biosynthetic\_gene\_cluster | 33 | 51.0272071072 | 472 | 2.4e-132 |
| CAQ18828.1 | polyketide\_synthase | BGC0000954 | Ajudazol\_biosynthetic\_gene\_cluster | 36 | 50.2498611882 | 472 | 3.1e-132 |
| CAD89777.1 | MelF\_protein | BGC0001010 | Melithiazol\_biosynthetic\_gene\_cluster | 33 | 50.9161576902 | 471 | 5.3e-132 |
| AEU11005.1 | NpnA | BGC0001029 | Nostophycin\_biosynthetic\_gene\_cluster | 33 | 52.6374236535 | 470 | 6.9e-132 |
| AMB48442.1 | polyketide\_synthase | BGC0001357 | Carbamidocyclophane\_biosynthetic\_gene\_cluster | 34 | 50.4719600222 | 468 | 4.5e-131 |
| AAW03329.1 | CtaF | BGC0000982 | Cystothiazole\_A\_biosynthetic\_gene\_cluster | 34 | 50.8606329817 | 467 | 5.8e-131 |
| AAO62584.1 | polyketide\_synthase\_type\_1 | BGC0001016 | Microcystin\_biosynthetic\_gene\_cluster | 32 | 58.856191005 | 467 | 7.6e-131 |
| BAV19379.1 | polyketide\_synthase | BGC0001390 | LL-Z1272beta\_biosynthetic\_gene\_cluster | 28 | 76.8461965575 | 467 | 7.6e-131 |
| CAQ18833.1 | polyketide\_synthase | BGC0000954 | Ajudazol\_biosynthetic\_gene\_cluster | 35 | 49.7501388118 | 466 | 1.3e-130 |
| AFV96138.1 | polyketide\_synthase | BGC0001064 | Cylindrocyclophane\_biosynthetic\_gene\_cluster | 34 | 50.1943364797 | 465 | 2.2e-130 |
| CBD77748.1 | polyketide\_synthase | BGC0000974 | Crocacin\_biosynthetic\_gene\_cluster | 33 | 55.9133814547 | 465 | 3.8e-130 |
| AAT70107.1 | CurL | BGC0001165 | Curacin\_biosynthetic\_gene\_cluster | 32 | 56.7462520822 | 463 | 1.1e-129 |
| AEE88278.1 | CurL | BGC0000976 | Curacin\_biosynthetic\_gene\_cluster | 32 | 56.7462520822 | 463 | 1.1e-129 |
| ADF88280.1 | polyketide\_synthase | BGC0000981 | Cylindrospermopsin\_biosynthetic\_gene\_cluster | 35 | 50.0277623542 | 463 | 1.4e-129 |
| CDM36726.1 | Beta-ketoacyl\_synthase | BGC0001360 | Mycophenolic\_acid\_biosynthetic\_gene\_cluster | 26 | 87.9511382565 | 462 | 1.9e-129 |
| AAF26921.1 | polyketide\_synthase | BGC0000988 | Epothilone\_biosynthetic\_gene\_cluster | 30 | 65.7967795669 | 462 | 2.4e-129 |
| ABX60162.1 | polyketide\_synthase | BGC0000978 | Cylindrospermopsin\_biosynthetic\_gene\_cluster | 35 | 50.0277623542 | 462 | 3.2e-129 |
| AAT70106.1 | CurK | BGC0001165 | Curacin\_biosynthetic\_gene\_cluster | 33 | 51.7490283176 | 461 | 4.2e-129 |
| AGC45624.1 | polyketide\_synthase | BGC0001394 | Phenalamide\_biosynthetic\_gene\_cluster | 30 | 70.3498056635 | 461 | 4.2e-129 |
| AEE88279.1 | CurK | BGC0000976 | Curacin\_biosynthetic\_gene\_cluster | 33 | 51.7490283176 | 461 | 4.2e-129 |
| ACB46195.1 | polyketide\_synthase | BGC0000989 | Epothilone\_biosynthetic\_gene\_cluster | 30 | 65.7967795669 | 460 | 7.1e-129 |
| ADB12491.1 | EpoD | BGC0000990 | Epothilone\_biosynthetic\_gene\_cluster | 30 | 65.7967795669 | 460 | 7.1e-129 |
| AAF62883.1 | epoD | BGC0000991 | Epothilone\_biosynthetic\_gene\_cluster | 29 | 65.7967795669 | 460 | 7.1e-129 |
| ADB12492.1 | EpoE | BGC0000990 | Epothilone\_biosynthetic\_gene\_cluster | 34 | 55.8578567463 | 460 | 9.3e-129 |
| CAD19091.1 | StiG\_protein | BGC0000153 | Stigmatellin\_biosynthetic\_gene\_cluster | 32 | 57.6346474181 | 460 | 1.2e-128 |
| AAF62884.1 | EpoE | BGC0000991 | Epothilone\_biosynthetic\_gene\_cluster | 34 | 55.8578567463 | 460 | 1.2e-128 |
| AAS98783.1 | JamL | BGC0001001 | Jamaicamide\_biosynthetic\_gene\_cluster | 33 | 54.4697390339 | 459 | 2.1e-128 |
| CCE88379.1 | polyketide\_synthase | BGC0001034 | Pellasoren\_biosynthetic\_gene\_cluster | 31 | 65.8523042754 | 458 | 3.5e-128 |
| AAU04878.1 | polyketide\_synthase | BGC0000365 | Glycopeptidolipid\_biosynthetic\_gene\_cluster | 33 | 53.9144919489 | 458 | 3.5e-128 |
| AAF26922.1 | polyketide\_synthase | BGC0000988 | Epothilone\_biosynthetic\_gene\_cluster | 34 | 55.9133814547 | 458 | 4.6e-128 |
| AAF19813.1 | MtaE | BGC0001024 | Myxothiazol\_biosynthetic\_gene\_cluster | 34 | 52.3042754026 | 456 | 1.3e-127 |
| AHB82053.1 | polyketide\_synthase | BGC0001019 | Microsclerodermins\_biosynthetic\_gene\_cluster | 35 | 50.1943364797 | 455 | 2.3e-127 |
| ACB46196.1 | polyketide\_synthase | BGC0000989 | Epothilone\_biosynthetic\_gene\_cluster | 33 | 55.6357579123 | 454 | 6.7e-127 |
| AAF00959.1 | McyD | BGC0001017 | Microcystin\_biosynthetic\_gene\_cluster | 35 | 49.9167129373 | 453 | 8.7e-127 |
| AEE88277.1 | CurM | BGC0000976 | Curacin\_biosynthetic\_gene\_cluster | 33 | 50.1943364797 | 453 | 1.5e-126 |
| ACV42478.1 | polyketide\_synthase | BGC0000043 | Curacin\_A\_biosynthetic\_gene\_cluster | 33 | 50.1943364797 | 453 | 1.5e-126 |
| AAT70108.1 | CurM | BGC0001165 | Curacin\_biosynthetic\_gene\_cluster | 33 | 50.1943364797 | 453 | 1.5e-126 |
| CAD19086.1 | StiB\_protein | BGC0000153 | Stigmatellin\_biosynthetic\_gene\_cluster | 34 | 49.8611882288 | 451 | 3.3e-126 |
| AEE88281.1 | CurI | BGC0000976 | Curacin\_biosynthetic\_gene\_cluster | 33 | 50.6385341477 | 451 | 3.3e-126 |
| AAT70104.1 | CurI | BGC0001165 | Curacin\_biosynthetic\_gene\_cluster | 33 | 50.6385341477 | 451 | 3.3e-126 |
| ACR33078.1 | polyketide\_synthase | BGC0000017 | Anatoxin\_biosynthetic\_gene\_cluster | 33 | 50.2498611882 | 450 | 9.6e-126 |
| ANI24099.1 | polyketide\_synthase | BGC0001235 | Antalid\_biosynthetic\_gene\_cluster | 32 | 51.4714047751 | 448 | 3.7e-125 |
| AAS98782.1 | JamK | BGC0001001 | Jamaicamide\_biosynthetic\_gene\_cluster | 34 | 47.418101055 | 448 | 3.7e-125 |
| AFU82615.1 | polyketide\_synthase | BGC0000998 | Guadinomine\_biosynthetic\_gene\_cluster | 36 | 49.4725152693 | 448 | 4.8e-125 |
| AAW03328.1 | CtaE | BGC0000982 | Cystothiazole\_A\_biosynthetic\_gene\_cluster | 33 | 51.9156024431 | 448 | 4.8e-125 |
| AGC45621.1 | polyketide\_synthase | BGC0001394 | Phenalamide\_biosynthetic\_gene\_cluster | 33 | 51.6935036091 | 448 | 4.8e-125 |
| ACR33079.1 | polyketide\_synthase | BGC0000017 | Anatoxin\_biosynthetic\_gene\_cluster | 33 | 50.6940588562 | 446 | 1.4e-124 |
| CAO98850.1 | polyketide\_synthase\_AufG | BGC0000023 | Aurafuron\_biosynthetic\_gene\_cluster | 32 | 50.9161576902 | 446 | 1.8e-124 |
| CCE88376.1 | polyketide\_synthase | BGC0001034 | Pellasoren\_biosynthetic\_gene\_cluster | 34 | 50.8606329817 | 445 | 4e-124 |
| AIT55259.1 | GulB | BGC0000072 | Gulmirecin\_biosynthetic\_gene\_cluster | 36 | 52.470849528 | 444 | 5.3e-124 |
| CAO98879.1 | polyketide\_synthase\_AufD | BGC0000023 | Aurafuron\_biosynthetic\_gene\_cluster | 34 | 50.1388117712 | 444 | 6.9e-124 |
| AEE88289.1 | CurA | BGC0000976 | Curacin\_biosynthetic\_gene\_cluster | 33 | 52.1932259856 | 444 | 6.9e-124 |
| AAT70096.1 | CurA | BGC0001165 | Curacin\_biosynthetic\_gene\_cluster | 33 | 52.1932259856 | 444 | 6.9e-124 |
| CAD19090.1 | StiF\_protein | BGC0000153 | Stigmatellin\_biosynthetic\_gene\_cluster | 33 | 52.9705719045 | 444 | 6.9e-124 |
| CAD19092.1 | StiH\_protein | BGC0000153 | Stigmatellin\_biosynthetic\_gene\_cluster | 32 | 49.8056635203 | 441 | 5.8e-123 |
| AAK57188.1 | MxaD | BGC0001022 | Myxalamid\_biosynthetic\_gene\_cluster | 34 | 50.1388117712 | 439 | 2.2e-122 |
| CAL58685.1 | polyketide\_synthase | BGC0000149 | Spirangien\_biosynthetic\_gene\_cluster | 33 | 50.8606329817 | 438 | 2.9e-122 |
| CAJ46689.1 | polyketide\_synthase | BGC0000969 | Chondramide\_biosynthetic\_gene\_cluster | 33 | 54.3031649084 | 438 | 2.9e-122 |
| CAD89776.1 | MelE\_protein | BGC0001009 | Melithiazol\_biosynthetic\_gene\_cluster | 32 | 51.4714047751 | 438 | 4.9e-122 |
| AFU82617.1 | polyketide\_synthase | BGC0000998 | Guadinomine\_biosynthetic\_gene\_cluster | 34 | 50.1943364797 | 438 | 4.9e-122 |
| CAC20921.1 | PimS2\_protein | BGC0000125 | Pimaricin\_biosynthetic\_gene\_cluster | 33 | 50.5830094392 | 437 | 6.5e-122 |
| CAQ34919.1 | polyketide\_synthase | BGC0000986 | Dkxanthene\_biosynthetic\_gene\_cluster | 34 | 50.3053858967 | 437 | 6.5e-122 |
| AAF26923.1 | polyketide\_synthase | BGC0000988 | Epothilone\_biosynthetic\_gene\_cluster | 35 | 49.9167129373 | 437 | 8.4e-122 |
| AIT55263.1 | GulF | BGC0000072 | Gulmirecin\_biosynthetic\_gene\_cluster | 33 | 50.4719600222 | 436 | 1.1e-121 |
| ACR33077.1 | polyketide\_synthase | BGC0000017 | Anatoxin\_biosynthetic\_gene\_cluster | 33 | 50.3053858967 | 436 | 1.1e-121 |
| AAK19883.1 | soraphen\_polyketide\_synthase\_A | BGC0000147 | Soraphen\_biosynthetic\_gene\_cluster | 33 | 51.5269294836 | 436 | 1.1e-121 |
| ADB12493.1 | EpoF | BGC0000990 | Epothilone\_biosynthetic\_gene\_cluster | 35 | 49.9167129373 | 436 | 1.9e-121 |
| ADH04639.1 | TgaA | BGC0001051 | Thuggacin\_biosynthetic\_gene\_cluster | 32 | 51.6379789006 | 435 | 2.5e-121 |
| CAO98847.1 | polyketide\_synthase\_AufC | BGC0000023 | Aurafuron\_biosynthetic\_gene\_cluster | 33 | 50.5274847307 | 435 | 3.2e-121 |
| CAD29793.1 | polyketide\_synthase\_type\_I | BGC0001015 | Microcystin\_biosynthetic\_gene\_cluster | 33 | 49.9167129373 | 435 | 3.2e-121 |
| CAL58686.1 | polyketide\_synthase | BGC0000149 | Spirangien\_biosynthetic\_gene\_cluster | 33 | 51.4158800666 | 435 | 4.2e-121 |
| CAJ46690.1 | polyketide\_synthase | BGC0000969 | Chondramide\_biosynthetic\_gene\_cluster | 30 | 65.130483065 | 435 | 4.2e-121 |
| ACB46197.1 | polyketide\_synthase | BGC0000989 | Epothilone\_biosynthetic\_gene\_cluster | 35 | 49.9167129373 | 434 | 5.5e-121 |
| ADZ24997.1 | polyketide\_synthase | BGC0000380 | Leupyrrin\_biosynthetic\_gene\_cluster | 33 | 51.7490283176 | 434 | 5.5e-121 |
| AGC45622.1 | polyketide\_synthase | BGC0001394 | Phenalamide\_biosynthetic\_gene\_cluster | 33 | 50.0277623542 | 434 | 5.5e-121 |
| CQR60493.1 | Polyketide\_synthase,\_type\_I,\_modules:\_9\_and\_10 | BGC0001287 | Chaxamycin\_A\_/\_chaxamycin\_B\_/\_chaxamycin\_C\_/\_chaxamycin\_D | 32 | 53.8589672404 | 433 | 1.6e-120 |
| AHN85651.1 | Phn2 | BGC0000122 | Phenylnannolone\_A\_biosynthetic\_gene\_cluster | 33 | 50.2498611882 | 432 | 2.1e-120 |
| ADX66461.1 | ScnS2 | BGC0000108 | Natamycin\_biosynthetic\_gene\_cluster | 33 | 50.6940588562 | 432 | 2.7e-120 |
| CAQ43076.1 | polyketide\_synthase | BGC0000970 | Chondrochloren\_biosynthetic\_gene\_cluster | 32 | 50.3609106052 | 431 | 3.5e-120 |
| AAF62885.1 | EpoF | BGC0000991 | Epothilone\_biosynthetic\_gene\_cluster | 34 | 49.9167129373 | 431 | 3.5e-120 |
| CAO98849.1 | polyketide\_synthase\_AufF | BGC0000023 | Aurafuron\_biosynthetic\_gene\_cluster | 35 | 50.0277623542 | 431 | 3.5e-120 |
| CCE88381.1 | polyketide\_synthase | BGC0001034 | Pellasoren\_biosynthetic\_gene\_cluster | 33 | 53.3037201555 | 431 | 4.6e-120 |
| AAO62582.1 | polyketide\_synthase\_peptide\_sythetase\_fusion\_protein | BGC0001016 | Microcystin\_biosynthetic\_gene\_cluster | 33 | 50.9716823987 | 431 | 4.6e-120 |
| AAF19810.1 | MtaB | BGC0001024 | Myxothiazol\_biosynthetic\_gene\_cluster | 32 | 49.9722376458 | 430 | 7.9e-120 |
| ADZ24998.1 | polyketide\_synthase | BGC0000380 | Leupyrrin\_biosynthetic\_gene\_cluster | 33 | 50.5830094392 | 430 | 7.9e-120 |
| CCE88380.1 | polyketide\_synthase | BGC0001034 | Pellasoren\_biosynthetic\_gene\_cluster | 33 | 50.5830094392 | 430 | 1.3e-119 |
| AAF71766.1 | NysI | BGC0000115 | Nystatin\_biosynthetic\_gene\_cluster | 32 | 50.3609106052 | 430 | 1.3e-119 |
| AAQ82561.1 | FscA | BGC0000061 | FR-008\_biosynthetic\_gene\_cluster | 34 | 51.5269294836 | 429 | 2.3e-119 |
| CAD19089.1 | StiE\_protein | BGC0000153 | Stigmatellin\_biosynthetic\_gene\_cluster | 34 | 49.9722376458 | 429 | 2.3e-119 |
| AAF71776.1 | NysC | BGC0000115 | Nystatin\_biosynthetic\_gene\_cluster | 33 | 50.9161576902 | 429 | 2.3e-119 |
| CAL58684.1 | polyketide\_synthase | BGC0000149 | Spirangien\_biosynthetic\_gene\_cluster | 32 | 50.9161576902 | 428 | 3e-119 |
| WP\_042799407.1 | polyketide\_synthase | BGC0001283 | Arsenopolyketides\_biosynthetic\_gene\_cluster | 32 | 52.8595224875 | 428 | 3e-119 |
| AHH34186.1 | polyketide\_synthase | BGC0001161 | 1-heptadecene\_biosynthetic\_gene\_cluster | 32 | 52.3042754026 | 428 | 3.9e-119 |
| AAQ82566.1 | FscF | BGC0000061 | FR-008\_biosynthetic\_gene\_cluster | 33 | 50.1388117712 | 427 | 8.7e-119 |
| AFP87523.1 | type\_I\_polyketide\_synthase | BGC0001159 | Marinopyrrole\_biosynthetic\_gene\_cluster | 33 | 50.7495835647 | 425 | 3.3e-118 |
| AIT55260.1 | GulC | BGC0000072 | Gulmirecin\_biosynthetic\_gene\_cluster | 32 | 51.3603553581 | 425 | 4.3e-118 |
| CAQ18830.1 | polyketide\_synthase | BGC0000954 | Ajudazol\_biosynthetic\_gene\_cluster | 31 | 59.9111604664 | 425 | 4.3e-118 |
| CAQ34928.1 | polyketide\_synthase | BGC0000986 | Dkxanthene\_biosynthetic\_gene\_cluster | 33 | 52.359800111 | 424 | 5.7e-118 |
| ADZ24996.1 | polyketide\_synthase | BGC0000380 | Leupyrrin\_biosynthetic\_gene\_cluster | 34 | 50.6940588562 | 423 | 9.6e-118 |
| AHA12079.1 | polyketide\_synthase\_type\_1 | BGC0001172 | Chlorizidine\_A\_biosynthetic\_gene\_cluster | 33 | 50.4164353137 | 423 | 9.6e-118 |
| ABK32263.1 | AmbH | BGC0000014 | Ambruticin\_biosynthetic\_gene\_cluster | 33 | 52.8595224875 | 423 | 1.3e-117 |
| BAT51065.1 | type\_I\_polyketide\_synthase | BGC0001296 | Streptazone\_E\_biosynthetic\_gene\_cluster | 32 | 50.8606329817 | 423 | 1.3e-117 |
| BAQ25512.1 | type\_I\_polyketide\_synthase | BGC0001288 | Maklamicin\_biosynthetic\_gene\_cluster | 32 | 52.0266518601 | 423 | 1.6e-117 |
| ADM46356.1 | polyketide\_synthase | BGC0000106 | Naphthomycin\_biosynthetic\_gene\_cluster | 33 | 53.3037201555 | 423 | 1.6e-117 |
| ALD82522.1 | polyketide\_synthase | BGC0001212 | Nannocystin\_biosynthetic\_gene\_cluster | 32 | 50.9161576902 | 422 | 2.1e-117 |
| BAR73019.1 | putative\_PKS\_(KS-AT-DH-KR-ACP-TE) | BGC0001194 | Hitachimycin\_biosynthetic\_gene\_cluster | 34 | 49.6390893948 | 422 | 2.8e-117 |
| ADH04658.1 | TugB | BGC0001342 | Thuggacin\_biosynthetic\_gene\_cluster | 33 | 50.0277623542 | 422 | 2.8e-117 |
| CUW01177.1 | Type\_I\_polyketide\_synthase | BGC0001359\_c2 | PM100117\_/\_PM100118\_biosynthetic\_gene\_cluster | 32 | 50.1388117712 | 421 | 3.7e-117 |
| CAD55506.1 | putative\_type\_I\_polyketide\_synthase | BGC0000038 | Coelimycin\_biosynthetic\_gene\_cluster | 31 | 51.4714047751 | 421 | 3.7e-117 |
| AAK73501.1 | AmphI | BGC0000015 | Amphotericin\_biosynthetic\_gene\_cluster | 33 | 50.9716823987 | 421 | 4.8e-117 |
| ADH04641.1 | TgaC | BGC0001051 | Thuggacin\_biosynthetic\_gene\_cluster | 32 | 50.7495835647 | 421 | 6.2e-117 |
| BAT51066.1 | type\_I\_polyketide\_synthase | BGC0001296 | Streptazone\_E\_biosynthetic\_gene\_cluster | 32 | 51.0272071072 | 421 | 6.2e-117 |
| BAC68129.1 | modular\_polyketide\_synthase | BGC0000059 | Filipin\_biosynthetic\_gene\_cluster | 33 | 49.9167129373 | 420 | 8.2e-117 |
| BAR73020.1 | putative\_PKS\_(KS-AT-DH-KR-ACP-KS-AT-DH-KR-ACP-KS-AT-DH-KR-ACP) | BGC0001194 | Hitachimycin\_biosynthetic\_gene\_cluster | 32 | 50.2498611882 | 420 | 8.2e-117 |
| AHH99920.1 | PKS\_I | BGC0000002 | Aculeximycin\_biosynthetic\_gene\_cluster | 33 | 49.7501388118 | 420 | 8.2e-117 |
| BAC68126.1 | modular\_polyketide\_synthase | BGC0000059 | Filipin\_biosynthetic\_gene\_cluster | 34 | 50.0277623542 | 419 | 1.8e-116 |
| CBD77738.1 | polyketide\_synthase | BGC0000974 | Crocacin\_biosynthetic\_gene\_cluster | 33 | 50.1388117712 | 419 | 2.4e-116 |
| ADM46358.1 | polyketide\_synthase | BGC0000106 | Naphthomycin\_biosynthetic\_gene\_cluster | 32 | 49.9722376458 | 419 | 2.4e-116 |
| AKD43522.1 | Type\_I\_polyketide\_synthase | BGC0001409 | Dutomycin\_biosynthetic\_gene\_cluster | 33 | 50.3053858967 | 419 | 2.4e-116 |
| ACY06289.1 | type\_I\_polyketide\_synthase | BGC0001042 | Sanglifehrin\_A\_biosynthetic\_gene\_cluster | 30 | 65.0749583565 | 418 | 3.1e-116 |
| AGC45623.1 | polyketide\_synthase | BGC0001394 | Phenalamide\_biosynthetic\_gene\_cluster | 34 | 51.0827318157 | 418 | 3.1e-116 |
| AHA12078.1 | polyketide\_synthase\_type\_1 | BGC0001172 | Chlorizidine\_A\_biosynthetic\_gene\_cluster | 32 | 50.6940588562 | 418 | 4e-116 |
| AAK73502.1 | AmphJ | BGC0000015 | Amphotericin\_biosynthetic\_gene\_cluster | 33 | 50.4719600222 | 418 | 4e-116 |
| AEU17899.1 | putative\_type\_I\_PKS | BGC0001072 | Simocyclinone\_biosynthetic\_gene\_cluster | 34 | 52.1377012771 | 418 | 5.3e-116 |
| AEZ54379.1 | PieA6 | BGC0000124 | Piericidin\_A1\_biosynthetic\_gene\_cluster | 32 | 51.5824541921 | 417 | 6.9e-116 |
| CUW01175.1 | Type\_I\_polyketide\_synthase | BGC0001359 | PM100117\_/\_PM100118\_biosynthetic\_gene\_cluster | 32 | 51.0272071072 | 417 | 6.9e-116 |
| ALA62323.1 | methylphloroacetophenone\_synthase | BGC0001309 | Usnic\_acid\_biosynthetic\_gene\_cluster | 29 | 69.0727373681 | 417 | 9e-116 |
| ACR50785.1 | polyketide\_synthase | BGC0000163 | Tetronasin\_biosynthetic\_gene\_cluster | 32 | 50.3053858967 | 417 | 9e-116 |
| ACN69989.1 | polyketide\_synthase | BGC0000079 | Indanomycin\_biosynthetic\_gene\_cluster | 32 | 51.9711271516 | 416 | 1.2e-115 |
| AAQ84156.1 | Plm1 | BGC0000123 | Phoslactomycin\_B\_biosynthetic\_gene\_cluster | 32 | 49.8611882288 | 416 | 1.2e-115 |
| ABW96540.1 | type\_I\_modular\_polyketide\_synthase | BGC0000159 | Tautomycin\_biosynthetic\_gene\_cluster | 33 | 50.1388117712 | 416 | 2e-115 |
| AGC24270.1 | PrlP | BGC0001038 | Pyralomicin\_biosynthetic\_gene\_cluster | 33 | 54.4697390339 | 416 | 2e-115 |
| CAJ88176.1 | putative\_polyketide\_synthase\_B | BGC0000151 | Stambomycin\_biosynthetic\_gene\_cluster | 32 | 52.3042754026 | 416 | 2e-115 |
| AAK73514.1 | AmphC | BGC0000015 | Amphotericin\_biosynthetic\_gene\_cluster | 32 | 50.8606329817 | 415 | 4.5e-115 |
| ABK32291.1 | JerE | BGC0000080 | Jerangolid\_biosynthetic\_gene\_cluster | 31 | 50.5274847307 | 415 | 4.5e-115 |
| ABB88521.1 | polyketide\_synthase\_type\_I | BGC0000050 | E-837\_biosynthetic\_gene\_cluster | 33 | 50.1943364797 | 415 | 4.5e-115 |
| AKD43769.1 | HerA2 | BGC0001349 | Heronamide\_biosynthetic\_gene\_cluster | 32 | 50.5830094392 | 415 | 4.5e-115 |
| ACO94484.1 | polyketide\_synthase\_type\_I | BGC0000097 | ML-449\_biosynthetic\_gene\_cluster | 31 | 49.6390893948 | 413 | 1.3e-114 |
| AAK73503.1 | AmphK | BGC0000015 | Amphotericin\_biosynthetic\_gene\_cluster | 32 | 57.6346474181 | 412 | 2.2e-114 |
| BAQ25511.1 | type\_I\_polyketide\_synthase | BGC0001288 | Maklamicin\_biosynthetic\_gene\_cluster | 32 | 50.9716823987 | 411 | 3.8e-114 |
| AAG13918.1 | megalomicin\_6-deoxyerythronolide\_B\_synthase\_2 | BGC0000092 | Megalomicin\_biosynthetic\_gene\_cluster | 33 | 50.6940588562 | 411 | 5e-114 |
| AAQ82567.1 | FscE | BGC0000034 | Candicidin\_biosynthetic\_gene\_cluster | 32 | 50.0832870627 | 411 | 5e-114 |
| CAC20931.1 | PimS1\_protein | BGC0000125 | Pimaricin\_biosynthetic\_gene\_cluster | 32 | 50.9716823987 | 410 | 8.4e-114 |
| ACR50774.1 | polyketide\_synthase | BGC0000163 | Tetronasin\_biosynthetic\_gene\_cluster | 31 | 49.7501388118 | 410 | 1.1e-113 |
| BAQ25513.1 | type\_I\_polyketide\_synthase | BGC0001288 | Maklamicin\_biosynthetic\_gene\_cluster | 31 | 56.1354802887 | 410 | 1.1e-113 |
| AAC01713.1 | RifD | BGC0000136 | Rifamycin\_biosynthetic\_gene\_cluster | 31 | 50.9161576902 | 410 | 1.4e-113 |
| BAF02926.1 | type\_I\_polyketide\_synthase | BGC0000073 | Halstoctacosanolide\_biosynthetic\_gene\_cluster | 32 | 51.3603553581 | 409 | 1.9e-113 |
| BAO66539.1 | type\_I\_polyketide\_synthase | BGC0000042 | Cremimycin\_biosynthetic\_gene\_cluster | 32 | 49.5835646863 | 409 | 1.9e-113 |
| CAC22145.1 | putative\_type\_I\_polyketide\_synthase | BGC0000038 | Coelimycin\_biosynthetic\_gene\_cluster | 31 | 50.4719600222 | 409 | 1.9e-113 |
| CAD17792.1 | probable\_non\_ribosomal\_peptide\_synthetase\_protein | BGC0001363 | Ralsolamycin\_biosynthetic\_gene\_cluster | 33 | 50.4164353137 | 409 | 2.5e-113 |
| AAC01711.1 | RifB | BGC0000136 | Rifamycin\_biosynthetic\_gene\_cluster | 32 | 50.2498611882 | 409 | 2.5e-113 |
| BAG84248.1 | putative\_polyketide\_synthase | BGC0000257 | Prodigiosin\_biosynthetic\_gene\_cluster | 33 | 50.1943364797 | 408 | 3.2e-113 |
| AAA79984.2 | soraphen\_polyketide\_synthase\_B | BGC0000147 | Soraphen\_biosynthetic\_gene\_cluster | 32 | 50.1388117712 | 408 | 3.2e-113 |
| BAQ25481.1 | type\_I\_polyketide\_synthase | BGC0001288 | Maklamicin\_biosynthetic\_gene\_cluster | 31 | 51.1382565242 | 408 | 3.2e-113 |
| ABV83221.1 | CppI | BGC0000116 | Nystatin-like\_Pseudonocardia\_polyene\_biosynthetic\_gene\_cluster | 33 | 51.1937812326 | 408 | 3.2e-113 |
| CAL58687.1 | polyketide\_synthase | BGC0000149 | Spirangien\_biosynthetic\_gene\_cluster | 30 | 55.9133814547 | 408 | 3.2e-113 |
| ADU86004.1 | putative\_modular\_polyketide\_synthase | BGC0000165 | Tiacumicin\_B\_biosynthetic\_gene\_cluster | 33 | 50.4164353137 | 408 | 4.2e-113 |
| EFL02193.1 | amino\_acid\_adenylation\_domain-containing\_protein | BGC0000996 | Frontalamides\_biosynthetic\_gene\_cluster | 32 | 52.7484730705 | 408 | 5.5e-113 |
| CAD29794.1 | peptide\_synthetase | BGC0001015 | Microcystin\_biosynthetic\_gene\_cluster | 33 | 51.0272071072 | 407 | 7.1e-113 |
| CAQ52622.1 | type\_I\_polyketide\_synthase,\_modules\_4-5 | BGC0001066 | Kendomycin\_biosynthetic\_gene\_cluster | 32 | 50.4164353137 | 407 | 7.1e-113 |
| ADX66459.1 | ScnS4 | BGC0000108 | Natamycin\_biosynthetic\_gene\_cluster | 32 | 50.3609106052 | 407 | 9.3e-113 |
| ACY06287.1 | type\_I\_polyketide\_synthase | BGC0001042 | Sanglifehrin\_A\_biosynthetic\_gene\_cluster | 33 | 49.6390893948 | 407 | 9.3e-113 |
| BAG17643.1 | putative\_NRPS-type-I\_PKS\_fusion\_protein | BGC0001043 | SGR\_PTMs\_biosynthetic\_gene\_cluster | 33 | 50.8051082732 | 407 | 9.3e-113 |
| AAC01714.1 | RifE | BGC0000136 | Rifamycin\_biosynthetic\_gene\_cluster | 32 | 49.8611882288 | 407 | 9.3e-113 |
| ADX66472.1 | ScnS1 | BGC0000108 | Natamycin\_biosynthetic\_gene\_cluster | 31 | 53.4147695725 | 406 | 1.2e-112 |
| AAQ82568.1 | FscD | BGC0000034 | Candicidin\_biosynthetic\_gene\_cluster | 32 | 50.3609106052 | 406 | 1.2e-112 |
| ABW96541.1 | type\_I\_modular\_polyketide\_synthase | BGC0000159 | Tautomycin\_biosynthetic\_gene\_cluster | 32 | 50.1943364797 | 406 | 1.2e-112 |
| ADU86002.1 | putative\_modular\_polyketide\_synthase | BGC0000165 | Tiacumicin\_B\_biosynthetic\_gene\_cluster | 32 | 49.5280399778 | 406 | 1.2e-112 |
| BAF02922.1 | type\_I\_polyketide\_synthase | BGC0000073 | Halstoctacosanolide\_biosynthetic\_gene\_cluster | 32 | 50.5830094392 | 406 | 1.2e-112 |
| BAO66542.1 | type\_I\_polyketide\_synthase | BGC0000042 | Cremimycin\_biosynthetic\_gene\_cluster | 32 | 49.6390893948 | 406 | 2.1e-112 |
| AIT55262.1 | GulE | BGC0000072 | Gulmirecin\_biosynthetic\_gene\_cluster | 32 | 49.9722376458 | 406 | 2.1e-112 |
| AAZ77673.1 | ChlB1 | BGC0000036 | Chlorothricin\_biosynthetic\_gene\_cluster | 33 | 50.7495835647 | 406 | 2.1e-112 |
| BAQ25483.1 | type\_I\_polyketide\_synthase | BGC0001288 | Maklamicin\_biosynthetic\_gene\_cluster | 31 | 51.0827318157 | 405 | 2.7e-112 |
| AAW03325.1 | CtaB | BGC0000982 | Cystothiazole\_A\_biosynthetic\_gene\_cluster | 32 | 50.8606329817 | 405 | 3.5e-112 |
| AEZ54375.1 | PieA2 | BGC0000124 | Piericidin\_A1\_biosynthetic\_gene\_cluster | 32 | 50.2498611882 | 405 | 3.5e-112 |
| CBD77736.1 | polyketide\_synthase | BGC0000974 | Crocacin\_biosynthetic\_gene\_cluster | 32 | 50.4719600222 | 405 | 3.5e-112 |
| ADC45535.1 | modular\_polyketide\_synthase | BGC0000093 | Meilingmycin\_biosynthetic\_gene\_cluster | 32 | 50.5830094392 | 405 | 3.5e-112 |
| AHH99921.1 | PKS\_I | BGC0000002 | Aculeximycin\_biosynthetic\_gene\_cluster | 31 | 49.4725152693 | 404 | 4.6e-112 |
| AAF71767.1 | NysJ | BGC0000115 | Nystatin\_biosynthetic\_gene\_cluster | 33 | 50.8606329817 | 404 | 4.6e-112 |
| AAZ77693.1 | ChlA1 | BGC0000036 | Chlorothricin\_biosynthetic\_gene\_cluster | 32 | 49.8056635203 | 404 | 6.1e-112 |
| BAH02271.1 | polyketide\_synthase | BGC0000126 | Pladienolide\_biosynthetic\_gene\_cluster | 31 | 52.5263742365 | 404 | 7.9e-112 |
| CQR60495.1 | Polyketide\_synthase,\_type\_I,\_module\_7 | BGC0001287 | Chaxamycin\_A\_/\_chaxamycin\_B\_/\_chaxamycin\_C\_/\_chaxamycin\_D | 31 | 51.5824541921 | 404 | 7.9e-112 |
| ACY13415.1 | KR\_domain\_protein | BGC0001367 | Haliamide\_biosynthetic\_gene\_cluster | 31 | 50.3053858967 | 404 | 7.9e-112 |
| ADH04660.1 | TugD | BGC0001342 | Thuggacin\_biosynthetic\_gene\_cluster | 32 | 51.3603553581 | 404 | 7.9e-112 |
| AAY28227.1 | HbmAIII | BGC0000074 | Herbimycin\_biosynthetic\_gene\_cluster | 32 | 51.6379789006 | 404 | 7.9e-112 |
| CQR60496.1 | Polyketide\_synthase,\_type\_I,\_modules:\_4,\_5\_and\_6 | BGC0001287 | Chaxamycin\_A\_/\_chaxamycin\_B\_/\_chaxamycin\_C\_/\_chaxamycin\_D | 32 | 50.2498611882 | 403 | 1e-111 |
| CBF80428.1 | polyketide\_synthase,\_putative\_(JCVI) | BGC0000680 | Austinol/dehydroaustinol,\_locus\_1\_biosynthetic\_gene\_cluster | 32 | 50.4719600222 | 403 | 1.3e-111 |
| AHA38199.1 | GphF | BGC0000069 | Gephyronic\_acid\_biosynthetic\_gene\_cluster | 33 | 51.0272071072 | 403 | 1.8e-111 |
| AFI57005.1 | QmnA1 | BGC0000133 | Quartromicin\_biosynthetic\_gene\_cluster | 33 | 49.3059411438 | 403 | 1.8e-111 |
| AEC13079.1 | fosA | BGC0000060 | Fostriecin\_biosynthetic\_gene\_cluster | 32 | 50.5830094392 | 403 | 1.8e-111 |
| IF55\_RS32375 | beta-ketoacyl\_synthase | BGC0001348 | JBIR-100\_biosynthetic\_gene\_cluster | 31 | 51.0272071072 | 402 | 2.3e-111 |
| BAR73017.1 | putative\_PKS\_(KS-AT-KR-ACP-KS-AT-DH-KR-ACP) | BGC0001194 | Hitachimycin\_biosynthetic\_gene\_cluster | 32 | 49.9167129373 | 402 | 2.3e-111 |
| ABO15860.1 | polyketide\_synthase | BGC0000130 | Pyrrolomycin\_biosynthetic\_gene\_cluster | 33 | 50.4719600222 | 402 | 2.3e-111 |
| AAG23263.1 | polyketide\_synthase\_extender\_modules\_5-7 | BGC0000148 | Spinosad\_biosynthetic\_gene\_cluster | 32 | 50.4164353137 | 401 | 3.9e-111 |
| ADH04640.1 | TgaB | BGC0001051 | Thuggacin\_biosynthetic\_gene\_cluster | 32 | 51.6379789006 | 401 | 3.9e-111 |
| CAD89773.1 | MelB\_protein | BGC0001009 | Melithiazol\_biosynthetic\_gene\_cluster | 32 | 51.0827318157 | 401 | 3.9e-111 |
| CAC20919.1 | PimS4\_protein | BGC0000125 | Pimaricin\_biosynthetic\_gene\_cluster | 32 | 50.0832870627 | 401 | 5.1e-111 |
| AAQ84157.1 | Plm2-3 | BGC0000123 | Phoslactomycin\_B\_biosynthetic\_gene\_cluster | 32 | 51.7490283176 | 401 | 5.1e-111 |
| AAC01712.2 | RifC | BGC0000136 | Rifamycin\_biosynthetic\_gene\_cluster | 32 | 50.9161576902 | 401 | 6.7e-111 |
| CAM00064.1 | EryAII\_Erythromycin\_polyketide\_synthase\_modules\_3\_and\_4 | BGC0000055 | Erythromycin\_biosynthetic\_gene\_cluster | 31 | 51.5269294836 | 401 | 6.7e-111 |
| AEH42490.1 | polyketide\_synthase | BGC0000032 | Calcimycin\_biosynthetic\_gene\_cluster | 33 | 50.0832870627 | 401 | 6.7e-111 |
| AMK48247.1 | 6-methylsalicylic\_acid\_synthase | BGC0001351\_c2 | Cahuitamycins\_biosynthetic\_gene\_cluster | 32 | 50.7495835647 | 401 | 6.7e-111 |
| ACN64831.1 | PokM1 | BGC0001061 | Polyketomycin\_biosynthetic\_gene\_cluster | 32 | 50.3053858967 | 401 | 6.7e-111 |
| ACR50775.1 | polyketide\_synthase | BGC0000163 | Tetronasin\_biosynthetic\_gene\_cluster | 30 | 49.9167129373 | 401 | 6.7e-111 |
| ADH04657.1 | TugA | BGC0001342 | Thuggacin\_biosynthetic\_gene\_cluster | 33 | 48.8617434758 | 401 | 6.7e-111 |
| BAD08359.1 | polyketide\_synthase\_modules\_5-6 | BGC0000167 | Vicenistatin\_biosynthetic\_gene\_cluster | 31 | 50.4719600222 | 400 | 8.7e-111 |
| AAM70355.1 | CalO5 | BGC0000033 | Calicheamicin\_biosynthetic\_gene\_cluster | 31 | 50.1388117712 | 400 | 8.7e-111 |
| CBA11584.1 | polyketide\_synthase\_type\_I | BGC0001046 | Streptolydigin\_biosynthetic\_gene\_cluster | 32 | 51.3603553581 | 400 | 1.1e-110 |
| AEH42491.1 | polyketide\_synthase | BGC0000032 | Calcimycin\_biosynthetic\_gene\_cluster | 33 | 49.8056635203 | 399 | 1.5e-110 |
| AHH99925.1 | PKS\_I | BGC0000002 | Aculeximycin\_biosynthetic\_gene\_cluster | 32 | 51.3603553581 | 399 | 1.5e-110 |
| BAH02268.1 | polyketide\_synthase | BGC0000126 | Pladienolide\_biosynthetic\_gene\_cluster | 32 | 51.3048306496 | 399 | 1.5e-110 |
| BAD38874.1 | polyketide\_synthase | BGC0000111 | Neocarzilin\_biosynthetic\_gene\_cluster | 33 | 50.3053858967 | 399 | 1.9e-110 |
| AJW65409.1 | type\_I\_modular\_polyketide\_synthase | BGC0001195 | Nocardiopsin\_biosynthetic\_gene\_cluster | 32 | 50.9161576902 | 399 | 1.9e-110 |
| AAX98185.1 | polyketide\_synthase\_type\_I | BGC0000052 | ECO-02301\_biosynthetic\_gene\_cluster | 32 | 50.1943364797 | 399 | 1.9e-110 |
| AAO06918.1 | GdmAIII | BGC0000066 | Geldanamycin\_biosynthetic\_gene\_cluster | 32 | 51.4158800666 | 399 | 1.9e-110 |
| ADM46360.1 | polyketide\_synthase | BGC0000106 | Naphthomycin\_biosynthetic\_gene\_cluster | 32 | 51.0827318157 | 399 | 2.5e-110 |
| AKD43768.1 | HerA1 | BGC0001349 | Heronamide\_biosynthetic\_gene\_cluster | 31 | 50.3609106052 | 399 | 2.5e-110 |
| ABB86410.1 | GelC | BGC0000067 | Geldanamycin\_biosynthetic\_gene\_cluster | 32 | 51.4158800666 | 399 | 2.5e-110 |
| BAF02924.1 | type\_I\_polyketide\_synthase | BGC0000073 | Halstoctacosanolide\_biosynthetic\_gene\_cluster | 31 | 52.7484730705 | 398 | 3.3e-110 |
| ABV97155.1 | Acyl\_transferase | BGC0000137 | Rifamycin\_biosynthetic\_gene\_cluster | 32 | 50.6940588562 | 398 | 3.3e-110 |
| CAQ52626.1 | type\_I\_polyketide\_synthase,\_loading\_module\_and\_modules\_1-3 | BGC0001066 | Kendomycin\_biosynthetic\_gene\_cluster | 31 | 50.0277623542 | 398 | 3.3e-110 |
| AAQ82565.1 | FscB | BGC0000034 | Candicidin\_biosynthetic\_gene\_cluster | 32 | 54.4697390339 | 398 | 4.3e-110 |
| ctg1\_orf10 |  | BGC0000053 | Elaiophylin\_biosynthetic\_gene\_cluster | 32 | 50.5830094392 | 398 | 4.3e-110 |
| BAE93729.1 | type\_I\_polyketide\_synthase | BGC0000164 | Tetronomycin\_biosynthetic\_gene\_cluster | 31 | 51.3048306496 | 398 | 5.7e-110 |
| ALP32042.1 | CycB | BGC0001293 | Cyclizidine\_biosynthetic\_gene\_cluster | 31 | 52.8595224875 | 398 | 5.7e-110 |
| AGZ15474.1 | putative\_type\_I\_polyketide\_synthase | BGC0001036 | Polyoxypeptin\_biosynthetic\_gene\_cluster | 31 | 56.0244308717 | 397 | 7.4e-110 |
| AIT55264.1 | GulA | BGC0000072 | Gulmirecin\_biosynthetic\_gene\_cluster | 33 | 48.4175458079 | 397 | 7.4e-110 |
| AAC01710.1 | RifA | BGC0000136 | Rifamycin\_biosynthetic\_gene\_cluster | 31 | 50.3053858967 | 397 | 9.7e-110 |
| WP\_063764077.1 | hypothetical\_protein | BGC0001348 | JBIR-100\_biosynthetic\_gene\_cluster | 32 | 50.9716823987 | 397 | 9.7e-110 |
| AHA38201.1 | GphH | BGC0000069 | Gephyronic\_acid\_biosynthetic\_gene\_cluster | 33 | 52.0821765686 | 397 | 9.7e-110 |
| CAQ52624.1 | type\_I\_polyketide\_synthase,\_modules\_7-8 | BGC0001066 | Kendomycin\_biosynthetic\_gene\_cluster | 31 | 50.5274847307 | 397 | 9.7e-110 |
| ACY06286.1 | polyketide\_synthase | BGC0001042 | Sanglifehrin\_A\_biosynthetic\_gene\_cluster | 31 | 51.4158800666 | 397 | 9.7e-110 |
| AEC13069.1 | fosC | BGC0000060 | Fostriecin\_biosynthetic\_gene\_cluster | 32 | 50.7495835647 | 396 | 1.3e-109 |
| BAC57028.1 | protomycinolide\_IV\_synthase\_1 | BGC0000102 | Mycinamicin\_biosynthetic\_gene\_cluster | 32 | 54.4697390339 | 396 | 1.3e-109 |
| AEK75504.1 | type\_1\_polyketide\_synthase | BGC0000001 | Abyssomicin\_biosynthetic\_gene\_cluster | 32 | 50.9161576902 | 396 | 1.6e-109 |
| BAF02921.1 | type\_I\_polyketide\_synthase | BGC0000073 | Halstoctacosanolide\_biosynthetic\_gene\_cluster | 30 | 54.9139367018 | 396 | 1.6e-109 |
| ABV97151.1 | AMP-dependent\_synthetase\_and\_ligase | BGC0000137 | Rifamycin\_biosynthetic\_gene\_cluster | 32 | 51.3603553581 | 396 | 1.6e-109 |
| CAI94682.1 | putative\_polyketide\_synthase | BGC0000141 | Rubradirin\_biosynthetic\_gene\_cluster | 31 | 51.4714047751 | 396 | 1.6e-109 |
| ABJ97439.1 | MerC | BGC0001012 | Meridamycin\_biosynthetic\_gene\_cluster | 32 | 49.8611882288 | 396 | 2.2e-109 |
| BAJ16467.1 | polyketide\_synthase | BGC0000058 | FD-891\_biosynthetic\_gene\_cluster | 32 | 51.4158800666 | 396 | 2.2e-109 |
| BAH02269.1 | polyketide\_synthase | BGC0000126 | Pladienolide\_biosynthetic\_gene\_cluster | 32 | 51.3048306496 | 396 | 2.2e-109 |
| BAF02923.1 | type\_I\_polyketide\_synthase | BGC0000073 | Halstoctacosanolide\_biosynthetic\_gene\_cluster | 32 | 50.7495835647 | 395 | 2.8e-109 |
| AHH99919.1 | PKS\_I | BGC0000002 | Aculeximycin\_biosynthetic\_gene\_cluster | 31 | 49.4725152693 | 395 | 2.8e-109 |
| ACA99172.1 | Polyketide\_synthase | BGC0001160 | Nonadecene\_biosynthetic\_gene\_cluster | 32 | 49.9722376458 | 395 | 3.7e-109 |
| ACB46471.1 | polyketide\_synthase | BGC0000082 | Kijanimicin\_biosynthetic\_gene\_cluster | 31 | 50.9161576902 | 395 | 3.7e-109 |
| CCP20047.1 | divK\_protein | BGC0001119 | Divergolide\_biosynthetic\_gene\_cluster | 31 | 51.5269294836 | 395 | 3.7e-109 |
| ABV91286.1 | type\_I\_modular\_polyketide\_synthase | BGC0000158 | Tautomycetin\_biosynthetic\_gene\_cluster | 31 | 49.8056635203 | 395 | 3.7e-109 |
| BAD08360.1 | polyketide\_synthase\_modules\_7-8 | BGC0000167 | Vicenistatin\_biosynthetic\_gene\_cluster | 30 | 52.359800111 | 394 | 4.8e-109 |
| CQR60494.1 | Polyketide\_synthase,\_type\_I,\_module\_8 | BGC0001287 | Chaxamycin\_A\_/\_chaxamycin\_B\_/\_chaxamycin\_C\_/\_chaxamycin\_D | 31 | 53.6368684064 | 394 | 6.3e-109 |
| AFV96142.1 | polyketide\_synthase | BGC0001064 | Cylindrocyclophane\_biosynthetic\_gene\_cluster | 31 | 50.3053858967 | 394 | 6.3e-109 |
| ALP32043.1 | CycC | BGC0001293 | Cyclizidine\_biosynthetic\_gene\_cluster | 32 | 50.3053858967 | 394 | 6.3e-109 |
| ABI94379.1 | tautomycetin\_biosynthetic\_PKS | BGC0000157 | Tautomycetin\_biosynthetic\_gene\_cluster | 31 | 49.8056635203 | 394 | 6.3e-109 |
| AAS98781.1 | JamJ | BGC0001001 | Jamaicamide\_biosynthetic\_gene\_cluster | 30 | 54.9139367018 | 394 | 6.3e-109 |
| ADC79639.1 | TamAIII | BGC0001052 | Tirandamycin\_biosynthetic\_gene\_cluster | 32 | 52.9705719045 | 394 | 6.3e-109 |
| CQR60497.1 | Polyketide\_synthase,\_type\_I,\_modules:\_loading,\_1,\_2\_and\_3 | BGC0001287 | Chaxamycin\_A\_/\_chaxamycin\_B\_/\_chaxamycin\_C\_/\_chaxamycin\_D | 31 | 51.4158800666 | 394 | 8.2e-109 |
| ADU86003.1 | putative\_modular\_polyketide\_synthase | BGC0000165 | Tiacumicin\_B\_biosynthetic\_gene\_cluster | 32 | 49.5280399778 | 394 | 8.2e-109 |
| ADC45515.1 | modular\_polyketide\_synthase | BGC0000093 | Meilingmycin\_biosynthetic\_gene\_cluster | 33 | 50.5830094392 | 393 | 1.1e-108 |
| AAG13919.1 | megalomicin\_6-deoxyerythronolide\_B\_synthase\_3 | BGC0000092 | Megalomicin\_biosynthetic\_gene\_cluster | 32 | 52.803997779 | 393 | 1.1e-108 |
| CAE46851.1 | Type\_I\_modular\_polyketide\_synthase | BGC0000103 | Mycolactone\_biosynthetic\_gene\_cluster | 31 | 50.6940588562 | 393 | 1.1e-108 |
| ACN69988.1 | polyketide\_synthase | BGC0000079 | Indanomycin\_biosynthetic\_gene\_cluster | 32 | 51.5269294836 | 393 | 1.4e-108 |
| CAE46843.1 | Type\_I\_modular\_polyketide\_synthase | BGC0000103 | Mycolactone\_biosynthetic\_gene\_cluster | 31 | 50.6940588562 | 393 | 1.4e-108 |
| CUW01176.1 | Type\_I\_polyketide\_synthase | BGC0001359\_c2 | PM100117\_/\_PM100118\_biosynthetic\_gene\_cluster | 31 | 51.1382565242 | 393 | 1.8e-108 |
| AAQ90173.1 | polyketide\_synthase\_type\_I | BGC0000128 | Pyoluteorin\_biosynthetic\_gene\_cluster | 32 | 49.7501388118 | 393 | 1.8e-108 |
| AEZ53945.1 | polyketide\_synthase | BGC0000144 | Salinomycin\_biosynthetic\_gene\_cluster | 33 | 50.4719600222 | 393 | 1.8e-108 |
| CAQ43075.1 | polyketide\_synthase | BGC0000970 | Chondrochloren\_biosynthetic\_gene\_cluster | 32 | 50.9716823987 | 392 | 2.4e-108 |
| AAR16521.1 | RimA | BGC0000138 | Rimocidin\_biosynthetic\_gene\_cluster | 30 | 50.9161576902 | 392 | 3.1e-108 |
| ACO94483.1 | polyketide\_synthase\_type\_I | BGC0000097 | ML-449\_biosynthetic\_gene\_cluster | 31 | 52.0266518601 | 392 | 3.1e-108 |
| AFV30247.1 | polyketide\_synthase | BGC0000075 | Hygrocin\_biosynthetic\_gene\_cluster | 32 | 50.4719600222 | 392 | 3.1e-108 |
| ctg1\_orf255 |  | BGC0001200 | Lorneic\_acid\_A\_biosynthetic\_gene\_cluster | 31 | 51.8045530261 | 392 | 3.1e-108 |
| CAJ88186.1 | putative\_modular\_polyketide\_synthase | BGC0000151 | Stambomycin\_biosynthetic\_gene\_cluster | 32 | 51.3603553581 | 391 | 4.1e-108 |
| ACF35445.1 | MbcAI | BGC0000090 | Macbecin\_biosynthetic\_gene\_cluster | 32 | 50.3609106052 | 391 | 5.3e-108 |
| ACO94496.1 | polyketide\_synthase\_type\_I | BGC0000097 | ML-449\_biosynthetic\_gene\_cluster | 32 | 50.7495835647 | 391 | 5.3e-108 |
| AKD43763.1 | HerG | BGC0001349 | Heronamide\_biosynthetic\_gene\_cluster | 32 | 50.3053858967 | 391 | 5.3e-108 |
| ACO94456.1 | polyketide\_synthase\_type\_I | BGC0000029 | BE-14106\_biosynthetic\_gene\_cluster | 31 | 52.7484730705 | 391 | 5.3e-108 |
| AHH99926.1 | PKS\_I | BGC0000002 | Aculeximycin\_biosynthetic\_gene\_cluster | 31 | 50.9161576902 | 391 | 5.3e-108 |
| ACB37755.1 | putative\_type\_I\_polyketide\_synthase | BGC0000162 | Tetrocarcin\_A\_biosynthetic\_gene\_cluster | 32 | 50.9716823987 | 391 | 5.3e-108 |
| ACF35446.1 | MbcAII | BGC0000090 | Macbecin\_biosynthetic\_gene\_cluster | 32 | 50.6940588562 | 391 | 6.9e-108 |
| AAZ77696.1 | ChlA3 | BGC0000036 | Chlorothricin\_biosynthetic\_gene\_cluster | 32 | 50.1943364797 | 390 | 9e-108 |
| ABC84471.1 | NigAVII | BGC0000114 | Nigericin\_biosynthetic\_gene\_cluster | 30 | 51.1937812326 | 390 | 9e-108 |
| ABV97152.1 | Beta-ketoacyl\_synthase | BGC0000137 | Rifamycin\_biosynthetic\_gene\_cluster | 32 | 50.2498611882 | 390 | 9e-108 |
| AFI57007.1 | QmnA3 | BGC0000133 | Quartromicin\_biosynthetic\_gene\_cluster | 31 | 51.5269294836 | 390 | 1.2e-107 |
| ACO94471.1 | polyketide\_synthase\_type\_I | BGC0000029 | BE-14106\_biosynthetic\_gene\_cluster | 31 | 50.1943364797 | 390 | 1.2e-107 |
| AHF22854.1 | MarL | BGC0000091 | Marineosin\_biosynthetic\_gene\_cluster | 32 | 50.4719600222 | 390 | 1.2e-107 |
| ABI93779.1 | GdmPKS | BGC0000068 | Geldanamycin\_biosynthetic\_gene\_cluster | 30 | 52.470849528 | 389 | 1.5e-107 |
| CAA16183.1 | polyketide\_synthase | BGC0001063 | Undecylprodigiosin\_biosynthetic\_gene\_cluster | 33 | 49.2504164353 | 389 | 1.5e-107 |
| ACY13414.1 | amino\_acid\_adenylation\_domain\_protein | BGC0001367 | Haliamide\_biosynthetic\_gene\_cluster | 30 | 55.3026096613 | 389 | 1.5e-107 |
| AAZ94386.1 | modular\_polyketide\_synthase | BGC0000040 | Concanamycin\_A\_biosynthetic\_gene\_cluster | 32 | 50.7495835647 | 389 | 2e-107 |
| AAZ77697.1 | ChlA4 | BGC0000036 | Chlorothricin\_biosynthetic\_gene\_cluster | 31 | 55.9689061632 | 389 | 2e-107 |
| ACY06288.1 | type\_I\_polyketide\_synthase | BGC0001042 | Sanglifehrin\_A\_biosynthetic\_gene\_cluster | 31 | 50.1943364797 | 389 | 2.6e-107 |
| AEZ64503.1 | Herd | BGC0001065 | Herboxidiene\_biosynthetic\_gene\_cluster | 32 | 51.4158800666 | 389 | 2.6e-107 |
| WP\_043476516.1 | beta-ketoacyl\_synthase | BGC0001232 | Leucanicidin\_biosynthetic\_gene\_cluster | 32 | 50.4719600222 | 389 | 2.6e-107 |
| ADM46359.1 | polyketide\_synthase | BGC0000106 | Naphthomycin\_biosynthetic\_gene\_cluster | 31 | 50.1943364797 | 388 | 3.4e-107 |
| AAZ94388.1 | nodular\_polyketide\_synthase | BGC0000040 | Concanamycin\_A\_biosynthetic\_gene\_cluster | 31 | 50.9161576902 | 388 | 3.4e-107 |
| ADC45534.1 | modular\_polyketide\_synthase | BGC0000093 | Meilingmycin\_biosynthetic\_gene\_cluster | 31 | 50.6385341477 | 388 | 3.4e-107 |
| ACB46487.1 | polyketide\_synthase | BGC0000082 | Kijanimicin\_biosynthetic\_gene\_cluster | 32 | 50.9161576902 | 388 | 4.5e-107 |
| CAQ64686.1 | lasalocid\_modular\_polyketide\_synthase | BGC0000087 | Lasalocid\_biosynthetic\_gene\_cluster | 31 | 50.0832870627 | 388 | 4.5e-107 |
| CAQ43079.1 | polyketide\_synthase | BGC0000970 | Chondrochloren\_biosynthetic\_gene\_cluster | 32 | 51.1937812326 | 388 | 4.5e-107 |
| AMB48441.1 | polyketide\_synthase | BGC0001357 | Carbamidocyclophane\_biosynthetic\_gene\_cluster | 29 | 54.5252637424 | 388 | 5.9e-107 |
| AEP40940.1 | polyketide\_synthase\_type\_I | BGC0000021 | Apoptolidin\_biosynthetic\_gene\_cluster | 32 | 49.9167129373 | 388 | 5.9e-107 |
| AGI99482.1 | Type\_I\_polyketide\_synthase | BGC0001004 | Lobophorin\_biosynthetic\_gene\_cluster | 30 | 51.1382565242 | 388 | 5.9e-107 |
| AHH99923.1 | PKS\_I | BGC0000002 | Aculeximycin\_biosynthetic\_gene\_cluster | 30 | 49.4725152693 | 387 | 7.7e-107 |
| BAG85026.1 | putative\_polyketide\_synthase | BGC0000086 | Lasalocid\_biosynthetic\_gene\_cluster | 31 | 50.0832870627 | 387 | 1e-106 |
| AGI99497.1 | type\_I\_polyketide\_synthase | BGC0001004 | Lobophorin\_biosynthetic\_gene\_cluster | 30 | 50.3609106052 | 387 | 1e-106 |
| AAZ77694.1 | ChlA2 | BGC0000036 | Chlorothricin\_biosynthetic\_gene\_cluster | 31 | 50.9161576902 | 387 | 1e-106 |
| AGY62755.1 | EbeC | BGC0000051 | Ebelactone\_biosynthetic\_gene\_cluster | 31 | 50.4164353137 | 386 | 1.3e-106 |
| ADC79637.1 | TamAI | BGC0001052 | Tirandamycin\_biosynthetic\_gene\_cluster | 32 | 50.1943364797 | 386 | 1.3e-106 |
| ABB88534.1 | polyketide\_synthase\_type\_I | BGC0000049 | E-492\_/\_E-975\_biosynthetic\_gene\_cluster | 32 | 50.0277623542 | 386 | 1.3e-106 |
| CAQ52623.1 | type\_I\_polyketide\_synthase,\_module\_6 | BGC0001066 | Kendomycin\_biosynthetic\_gene\_cluster | 31 | 50.4164353137 | 386 | 1.7e-106 |
| BAQ21939.1 | putative\_type\_I\_polyketide\_synthase | BGC0001204 | Versipelostatin\_biosynthetic\_gene\_cluster | 32 | 51.0827318157 | 386 | 1.7e-106 |
| ABW96542.1 | type\_I\_modular\_polyketide\_synthase | BGC0000159 | Tautomycin\_biosynthetic\_gene\_cluster | 31 | 50.8606329817 | 386 | 1.7e-106 |
| AAZ77698.1 | ChlA5 | BGC0000036 | Chlorothricin\_biosynthetic\_gene\_cluster | 32 | 50.9716823987 | 386 | 2.2e-106 |
| AAF71774.1 | NysA | BGC0000115 | Nystatin\_biosynthetic\_gene\_cluster | 32 | 50.5830094392 | 386 | 2.2e-106 |
| ABB05104.1 | LipPks3 | BGC0001003 | Lipomycin\_biosynthetic\_gene\_cluster | 31 | 52.1932259856 | 386 | 2.2e-106 |
| AGC09499.1 | LobS4 | BGC0001183 | Lobophorin\_biosynthetic\_gene\_cluster | 30 | 51.1382565242 | 386 | 2.2e-106 |
| AFV30251.1 | polyketide\_synthase | BGC0000075 | Hygrocin\_biosynthetic\_gene\_cluster | 31 | 50.6940588562 | 386 | 2.2e-106 |
| ABB88523.1 | polyketide\_synthase\_type\_I | BGC0000050 | E-837\_biosynthetic\_gene\_cluster | 31 | 50.9716823987 | 385 | 3.8e-106 |
| ACN69990.1 | polyketide\_synthase | BGC0000079 | Indanomycin\_biosynthetic\_gene\_cluster | 31 | 50.4164353137 | 385 | 3.8e-106 |
| AEU17898.1 | putative\_type\_I\_PKS | BGC0001072 | Simocyclinone\_biosynthetic\_gene\_cluster | 30 | 52.470849528 | 385 | 3.8e-106 |
| ACO94468.1 | polyketide\_synthase\_type\_I | BGC0000029 | BE-14106\_biosynthetic\_gene\_cluster | 31 | 50.9716823987 | 385 | 3.8e-106 |
| CAE45670.1 | borrelidin\_polyketide\_synthase,\_type\_I | BGC0000031 | Borrelidin\_biosynthetic\_gene\_cluster | 31 | 49.4169905608 | 384 | 5e-106 |
| AAP42873.1 | NanA11 | BGC0000105 | Nanchangmycin\_biosynthetic\_gene\_cluster | 32 | 50.0832870627 | 384 | 5e-106 |
| AKD43761.1 | HerD | BGC0001349 | Heronamide\_biosynthetic\_gene\_cluster | 31 | 50.6940588562 | 384 | 5e-106 |
| AAZ94390.1 | modular\_polyketide\_synthase | BGC0000040 | Concanamycin\_A\_biosynthetic\_gene\_cluster | 31 | 50.3053858967 | 384 | 6.5e-106 |
| AAC46024.1 | polyketide\_synthase\_modules\_1\_and\_2 | BGC0000113 | Niddamycin\_biosynthetic\_gene\_cluster | 32 | 49.8611882288 | 384 | 8.5e-106 |
| CUW01174.1 | Type\_I\_polyketide\_synthase | BGC0001359 | PM100117\_/\_PM100118\_biosynthetic\_gene\_cluster | 31 | 51.1382565242 | 384 | 8.5e-106 |
| AFP87524.1 | type\_I\_polyketide\_synthase | BGC0001159 | Marinopyrrole\_biosynthetic\_gene\_cluster | 32 | 51.3048306496 | 384 | 8.5e-106 |
| AHH99922.1 | PKS\_I | BGC0000002 | Aculeximycin\_biosynthetic\_gene\_cluster | 30 | 49.4725152693 | 384 | 8.5e-106 |
| CBA11583.1 | polyketide\_synthase\_type\_I | BGC0001046 | Streptolydigin\_biosynthetic\_gene\_cluster | 30 | 51.1382565242 | 383 | 1.4e-105 |
| BAV32159.1 | polyketide\_synthase | BGC0001373 | Sordarin\_biosynthetic\_gene\_cluster | 32 | 51.4158800666 | 383 | 1.4e-105 |
| CAQ64691.1 | lasalocid\_modular\_polyketide\_synthase | BGC0000087 | Lasalocid\_biosynthetic\_gene\_cluster | 30 | 50.7495835647 | 383 | 1.4e-105 |
| ABV83222.1 | CppJ | BGC0000116 | Nystatin-like\_Pseudonocardia\_polyene\_biosynthetic\_gene\_cluster | 32 | 51.0272071072 | 383 | 1.4e-105 |
| ABI91466.1 | beta-ketoacyl\_synthase | BGC0001094 | Enacyloxin\_biosynthetic\_gene\_cluster | 32 | 50.1388117712 | 383 | 1.9e-105 |
| AAF86396.1 | FkbA | BGC0000994 | FK520\_biosynthetic\_gene\_cluster | 31 | 50.5830094392 | 383 | 1.9e-105 |
| AEZ54374.1 | PieA1 | BGC0000124 | Piericidin\_A1\_biosynthetic\_gene\_cluster | 30 | 51.2493059411 | 383 | 1.9e-105 |
| ctg1\_orf11 |  | BGC0000053 | Elaiophylin\_biosynthetic\_gene\_cluster | 31 | 51.5824541921 | 383 | 1.9e-105 |
| CAE02605.1 | polyketide\_synthase\_type\_I | BGC0000024 | Aureothin\_biosynthetic\_gene\_cluster | 31 | 50.3053858967 | 382 | 2.5e-105 |
| CAE45671.1 | borrelidin\_polyketide\_synthase,\_type\_I | BGC0000031 | Borrelidin\_biosynthetic\_gene\_cluster | 30 | 53.5258189895 | 382 | 2.5e-105 |
| ALP32046.1 | CycF | BGC0001293 | Cyclizidine\_biosynthetic\_gene\_cluster | 31 | 50.4719600222 | 382 | 3.2e-105 |
| ctg1\_orf7 |  | BGC0000053 | Elaiophylin\_biosynthetic\_gene\_cluster | 33 | 50.3609106052 | 381 | 4.2e-105 |
| CUW01172.1 | Type\_I\_polyketide\_synthase | BGC0001359 | PM100117\_/\_PM100118\_biosynthetic\_gene\_cluster | 30 | 53.1926707385 | 381 | 4.2e-105 |
| AHE80996.1 | PieA6 | BGC0001169 | Piericidin\_A1\_biosynthetic\_gene\_cluster | 31 | 51.2493059411 | 381 | 4.2e-105 |
| BAC57032.1 | protomycinolide\_IV\_synthase\_5 | BGC0000102 | Mycinamicin\_biosynthetic\_gene\_cluster | 32 | 51.8045530261 | 381 | 4.2e-105 |
| WP\_048832936.1 | polyketide\_synthase | BGC0001348 | JBIR-100\_biosynthetic\_gene\_cluster | 31 | 52.5263742365 | 381 | 5.5e-105 |
| CAC20920.1 | PimS3\_protein | BGC0000125 | Pimaricin\_biosynthetic\_gene\_cluster | 32 | 50.0277623542 | 381 | 5.5e-105 |
| AAK73513.1 | AmphB | BGC0000015 | Amphotericin\_biosynthetic\_gene\_cluster | 31 | 50.5830094392 | 381 | 7.2e-105 |
| BAB69192.1 | modular\_polyketide\_synthase | BGC0000117 | Oligomycin\_biosynthetic\_gene\_cluster | 32 | 50.1388117712 | 381 | 7.2e-105 |
| AAB66507.1 | tylactone\_synthase\_module\_6 | BGC0000166 | Tylactone\_biosynthetic\_gene\_cluster | 32 | 51.4714047751 | 380 | 9.4e-105 |
| BAG85031.1 | putative\_polyketide\_synthase | BGC0000086 | Lasalocid\_biosynthetic\_gene\_cluster | 30 | 51.1382565242 | 380 | 9.4e-105 |
| AAC38075.1 | polyketide\_synthase\_type\_I | BGC0000127 | Pyoluteorin\_biosynthetic\_gene\_cluster | 32 | 50.6940588562 | 380 | 1.2e-104 |
| ADC79638.1 | TamAII | BGC0001052 | Tirandamycin\_biosynthetic\_gene\_cluster | 29 | 54.9694614103 | 380 | 1.2e-104 |
| CAQ64692.1 | lasalocid\_modular\_polyketide\_synthase | BGC0000087 | Lasalocid\_biosynthetic\_gene\_cluster | 30 | 52.5263742365 | 380 | 1.2e-104 |
| BAQ25507.1 | type\_I\_polyketide\_synthase | BGC0001288 | Maklamicin\_biosynthetic\_gene\_cluster | 32 | 49.9722376458 | 379 | 2.1e-104 |
| CUW01171.1 | Type\_I\_polyketide\_synthase | BGC0001359 | PM100117\_/\_PM100118\_biosynthetic\_gene\_cluster | 30 | 51.5269294836 | 379 | 2.1e-104 |
| ABB88535.1 | polyketide\_synthase\_type\_I | BGC0000049\_c2 | E-492\_/\_E-975\_biosynthetic\_gene\_cluster | 30 | 55.4136590783 | 379 | 2.7e-104 |
| CAE45669.1 | borrelidin\_polyketide\_synthase,\_type\_I | BGC0000031 | Borrelidin\_biosynthetic\_gene\_cluster | 31 | 49.5835646863 | 378 | 3.6e-104 |
| CAC20930.1 | PimS0\_protein | BGC0000125 | Pimaricin\_biosynthetic\_gene\_cluster | 31 | 50.4164353137 | 378 | 3.6e-104 |
| ABV83230.1 | CppC | BGC0000116 | Nystatin-like\_Pseudonocardia\_polyene\_biosynthetic\_gene\_cluster | 31 | 50.3609106052 | 378 | 3.6e-104 |
| ABV97153.1 | Beta-ketoacyl\_synthase | BGC0000137 | Rifamycin\_biosynthetic\_gene\_cluster | 31 | 50.3053858967 | 378 | 4.6e-104 |
| CAQ18838.1 | polyketide\_synthase | BGC0000954 | Ajudazol\_biosynthetic\_gene\_cluster | 33 | 49.7501388118 | 378 | 4.6e-104 |
| ABC87509.1 | polyketide\_synthase | BGC0001011 | Meridamycin\_biosynthetic\_gene\_cluster | 31 | 50.5830094392 | 378 | 6.1e-104 |
| ABC84469.1 | NigAIX | BGC0000114 | Nigericin\_biosynthetic\_gene\_cluster | 30 | 55.1915602443 | 378 | 6.1e-104 |
| AEZ54377.1 | PieA4 | BGC0000124 | Piericidin\_A1\_biosynthetic\_gene\_cluster | 30 | 49.8611882288 | 378 | 6.1e-104 |
| ABY66019.1 | 6-methylsalicylic\_acid\_synthase | BGC0001008 | Maduropeptin\_biosynthetic\_gene\_cluster | 33 | 50.6385341477 | 378 | 6.1e-104 |
| CAJ88187.2 | putative\_type\_I\_modular\_polyketide\_synthase | BGC0000151 | Stambomycin\_biosynthetic\_gene\_cluster | 32 | 50.7495835647 | 378 | 6.1e-104 |
| CAJ88177.1 | putative\_type\_I\_polyketide\_synthase | BGC0000151 | Stambomycin\_biosynthetic\_gene\_cluster | 32 | 49.8611882288 | 378 | 6.1e-104 |
| ctg1\_orf20 |  | BGC0001013 | Meridamycin\_biosynthetic\_gene\_cluster | 31 | 50.5830094392 | 378 | 6.1e-104 |
| AHB82063.1 | polyketide\_synthase | BGC0001231 | Microsclerodermins\_biosynthetic\_gene\_cluster | 36 | 38.8117712382 | 377 | 7.9e-104 |
| AJW65410.1 | type\_I\_modular\_polyketide\_synthase | BGC0001195 | Nocardiopsin\_biosynthetic\_gene\_cluster | 31 | 54.2476401999 | 377 | 1e-103 |
| AAK73512.1 | AmphA | BGC0000015 | Amphotericin\_biosynthetic\_gene\_cluster | 32 | 52.0266518601 | 377 | 1e-103 |
| ABB86408.1 | GelA | BGC0000067 | Geldanamycin\_biosynthetic\_gene\_cluster | 30 | 51.0272071072 | 377 | 1e-103 |
| AAO06916.1 | GdmAI | BGC0000066 | Geldanamycin\_biosynthetic\_gene\_cluster | 30 | 51.1382565242 | 376 | 1.4e-103 |
| BAF02925.1 | type\_I\_polyketide\_synthase | BGC0000073 | Halstoctacosanolide\_biosynthetic\_gene\_cluster | 30 | 50.7495835647 | 376 | 1.4e-103 |
| ADX66470.1 | ScnS0 | BGC0000108 | Natamycin\_biosynthetic\_gene\_cluster | 30 | 50.9161576902 | 376 | 1.8e-103 |
| BAD08373.1 | polyketide\_synthase\_modules\_1-3 | BGC0000167 | Vicenistatin\_biosynthetic\_gene\_cluster | 30 | 50.1388117712 | 376 | 2.3e-103 |
| CCP20051.1 | divM\_protein | BGC0001119 | Divergolide\_biosynthetic\_gene\_cluster | 31 | 51.0827318157 | 376 | 2.3e-103 |
| CAJ88184.1 | putative\_modular\_polyketide\_synthase | BGC0000151 | Stambomycin\_biosynthetic\_gene\_cluster | 32 | 49.7501388118 | 375 | 3e-103 |
| ctg1\_orf521 |  | BGC0001199 | Akaeolide\_biosynthetic\_gene\_cluster | 31 | 50.0832870627 | 375 | 3e-103 |
| ADX66460.1 | ScnS3 | BGC0000108 | Natamycin\_biosynthetic\_gene\_cluster | 32 | 50.3053858967 | 375 | 3e-103 |
| BAO66541.1 | type\_I\_polyketide\_synthase | BGC0000042 | Cremimycin\_biosynthetic\_gene\_cluster | 31 | 49.6946141033 | 375 | 3.9e-103 |
| CAJ88185.2 | putative\_modular\_polyketide\_synthase | BGC0000151 | Stambomycin\_biosynthetic\_gene\_cluster | 31 | 51.1382565242 | 375 | 3.9e-103 |
| AHE80994.1 | PieA4 | BGC0001169 | Piericidin\_A1\_biosynthetic\_gene\_cluster | 31 | 49.6946141033 | 374 | 5.1e-103 |
| CAN89636.1 | putative\_polyketide\_synthase | BGC0001070 | Kirromycin\_biosynthetic\_gene\_cluster | 30 | 50.4164353137 | 374 | 5.1e-103 |
| ACB46488.1 | polyketide\_synthase | BGC0000082 | Kijanimicin\_biosynthetic\_gene\_cluster | 30 | 50.4164353137 | 374 | 6.7e-103 |
| AAP42874.1 | NanA8 | BGC0000105 | Nanchangmycin\_biosynthetic\_gene\_cluster | 30 | 52.915047196 | 374 | 8.8e-103 |
| AGC24271.1 | PrlQ | BGC0001038 | Pyralomicin\_biosynthetic\_gene\_cluster | 32 | 52.3042754026 | 373 | 1.1e-102 |
| ALP32045.1 | CycE | BGC0001293 | Cyclizidine\_biosynthetic\_gene\_cluster | 30 | 52.0821765686 | 373 | 1.5e-102 |
| ctg1\_orf0002 |  | BGC0001068 | Pyripyropene\_A\_biosynthetic\_gene\_cluster | 31 | 53.026096613 | 373 | 1.5e-102 |
| ABC84470.1 | NIGAVIII | BGC0000114 | Nigericin\_biosynthetic\_gene\_cluster | 29 | 53.248195447 | 373 | 1.5e-102 |
| AGI99496.1 | Type\_I\_polyketide\_synthase | BGC0001004 | Lobophorin\_biosynthetic\_gene\_cluster | 30 | 49.9722376458 | 373 | 1.5e-102 |
| ctg1\_orf253 |  | BGC0001200 | Lorneic\_acid\_A\_biosynthetic\_gene\_cluster | 33 | 50.5274847307 | 373 | 2e-102 |
| BAC68127.1 | modular\_polyketide\_synthase | BGC0000059 | Filipin\_biosynthetic\_gene\_cluster | 30 | 53.0816213215 | 373 | 2e-102 |
| ABC84456.1 | NigAI | BGC0000114 | Nigericin\_biosynthetic\_gene\_cluster | 31 | 52.4153248195 | 373 | 2e-102 |
| CAD15508.1 | probable\_polyketide\_synthase\_protein | BGC0001014 | Micacocidin\_biosynthetic\_gene\_cluster | 33 | 50.9161576902 | 372 | 3.3e-102 |
| ANC94966.1 | AlmHI | BGC0001396 | Aldgamycin\_biosynthetic\_gene\_cluster | 31 | 50.7495835647 | 372 | 3.3e-102 |
| AAP85335.1 | type\_I\_PKS | BGC0000233 | Hedamycin\_biosynthetic\_gene\_cluster | 30 | 49.8611882288 | 372 | 3.3e-102 |
| AEK75502.1 | type\_1\_polyketide\_synthase | BGC0000001 | Abyssomicin\_biosynthetic\_gene\_cluster | 32 | 49.9167129373 | 372 | 3.3e-102 |
| AAP42859.1 | NanA5 | BGC0000105 | Nanchangmycin\_biosynthetic\_gene\_cluster | 30 | 50.4164353137 | 371 | 4.3e-102 |
| AAZ94387.1 | modular\_polyketide\_synthase | BGC0000040 | Concanamycin\_A\_biosynthetic\_gene\_cluster | 31 | 51.3048306496 | 371 | 4.3e-102 |
| ACN69992.1 | polyketide\_synthase | BGC0000079 | Indanomycin\_biosynthetic\_gene\_cluster | 30 | 51.5269294836 | 371 | 5.7e-102 |
| BAB69195.1 | modular\_polyketide\_synthase | BGC0000117 | Oligomycin\_biosynthetic\_gene\_cluster | 31 | 51.2493059411 | 371 | 7.4e-102 |
| CUW01191.1 | Type\_I\_polyketide\_synthase | BGC0001359\_c2 | PM100117\_/\_PM100118\_biosynthetic\_gene\_cluster | 30 | 51.3048306496 | 371 | 7.4e-102 |
| ABY83164.1 | Azi26 | BGC0000960 | Azinomycin\_B\_biosynthetic\_gene\_cluster | 32 | 50.5830094392 | 371 | 7.4e-102 |
| ABB86409.1 | GelB | BGC0000067 | Geldanamycin\_biosynthetic\_gene\_cluster | 30 | 51.5824541921 | 371 | 7.4e-102 |
| AIA58899.1 | HRPKS | BGC0001141 | Brefeldin\_biosynthetic\_gene\_cluster | 31 | 54.8584119933 | 371 | 7.4e-102 |
| AAG23262.1 | polyketide\_synthase\_extender\_modules\_8-10 | BGC0000148 | Spinosad\_biosynthetic\_gene\_cluster | 30 | 51.1937812326 | 370 | 9.7e-102 |
| CAM00065.1 | EryAIII\_Erythromycin\_polyketide\_synthase\_modules\_5\_and\_6 | BGC0000055 | Erythromycin\_biosynthetic\_gene\_cluster | 30 | 51.8600777346 | 370 | 9.7e-102 |
| CAL58681.1 | polyketide\_synthase | BGC0000149 | Spirangien\_biosynthetic\_gene\_cluster | 31 | 48.4730705164 | 370 | 9.7e-102 |
| CAJ88175.1 | putative\_polyketide\_synthase\_B | BGC0000151 | Stambomycin\_biosynthetic\_gene\_cluster | 30 | 50.4164353137 | 370 | 9.7e-102 |
| ACB37741.1 | putative\_type\_I\_polyketide\_synthase | BGC0000162 | Tetrocarcin\_A\_biosynthetic\_gene\_cluster | 30 | 50.9161576902 | 370 | 9.7e-102 |
| AEU17897.1 | putative\_type\_I\_PKS | BGC0001072 | Simocyclinone\_biosynthetic\_gene\_cluster | 30 | 51.0272071072 | 370 | 1.3e-101 |
| EAL89230.2 | LovB-like\_polyketide\_synthase,\_putative | BGC0000129 | Pyripyropene\_biosynthetic\_gene\_cluster | 31 | 51.5824541921 | 369 | 2.2e-101 |
| AAY28226.1 | HbmAII | BGC0000074 | Herbimycin\_biosynthetic\_gene\_cluster | 31 | 51.7490283176 | 369 | 2.2e-101 |
| EHA28244.1 | hypothetical\_protein | BGC0001143 | Azanigerone\_biosynthetic\_gene\_cluster | 30 | 52.359800111 | 369 | 2.2e-101 |
| ctg1\_orf522 |  | BGC0001199 | Akaeolide\_biosynthetic\_gene\_cluster | 31 | 51.8600777346 | 369 | 2.8e-101 |
| BI06\_RS39075 | polyketide\_synthase | BGC0001232 | Leucanicidin\_biosynthetic\_gene\_cluster | 31 | 50.8606329817 | 369 | 2.8e-101 |
| ABV83223.1 | CppK | BGC0000116 | Nystatin-like\_Pseudonocardia\_polyene\_biosynthetic\_gene\_cluster | 31 | 54.5807884509 | 369 | 2.8e-101 |
| AGY30677.1 | Ann5 | BGC0001298 | 4-Z-annimycin\_biosynthetic\_gene\_cluster | 31 | 51.4714047751 | 369 | 2.8e-101 |
| CAD19085.1 | StiA\_protein | BGC0000153 | Stigmatellin\_biosynthetic\_gene\_cluster | 33 | 49.2504164353 | 368 | 3.7e-101 |
| ABC87510.1 | polyketide\_synthase | BGC0001011 | Meridamycin\_biosynthetic\_gene\_cluster | 31 | 50.3609106052 | 368 | 3.7e-101 |
| AAS98787.1 | JamP | BGC0001001 | Jamaicamide\_biosynthetic\_gene\_cluster | 32 | 50.5274847307 | 368 | 3.7e-101 |
| ABC84460.1 | NigAV | BGC0000114 | Nigericin\_biosynthetic\_gene\_cluster | 30 | 51.8045530261 | 368 | 3.7e-101 |
| ctg1\_orf21 |  | BGC0001013 | Meridamycin\_biosynthetic\_gene\_cluster | 31 | 50.3609106052 | 368 | 3.7e-101 |
| ACO94499.1 | polyketide\_synthase\_type\_I | BGC0000097 | ML-449\_biosynthetic\_gene\_cluster | 30 | 51.7490283176 | 368 | 3.7e-101 |
| BAC76491.1 | lankamycin\_synthase\_LkmAIII | BGC0000085 | Lankamycin\_biosynthetic\_gene\_cluster | 30 | 50.7495835647 | 368 | 4.8e-101 |
| AAO65796.1 | monensin\_polyketide\_synthase\_loading\_module\_and\_module\_1 | BGC0000100 | Monensin\_biosynthetic\_gene\_cluster | 32 | 52.470849528 | 368 | 4.8e-101 |
| AEZ54378.1 | PieA5 | BGC0000124 | Piericidin\_A1\_biosynthetic\_gene\_cluster | 31 | 50.9161576902 | 368 | 4.8e-101 |
| AAM77986.1 | iterative\_type\_I\_polyketide\_synthase | BGC0000112 | Neocarzinostatin\_biosynthetic\_gene\_cluster | 31 | 49.7501388118 | 368 | 6.3e-101 |
| AEZ64505.1 | Herb | BGC0001065 | Herboxidiene\_biosynthetic\_gene\_cluster | 31 | 52.0821765686 | 367 | 8.2e-101 |
| BAK64649.1 | polyketide\_synthase | BGC0000135 | Reveromycin\_biosynthetic\_gene\_cluster | 32 | 50.4164353137 | 367 | 1.1e-100 |
| BAC76492.1 | lankamycin\_synthase\_LkmAII | BGC0000085 | Lankamycin\_biosynthetic\_gene\_cluster | 31 | 50.5830094392 | 366 | 1.4e-100 |
| AJO72736.1 | Type\_I\_modular\_polyketide\_synthase | BGC0001381 | Brasilinolide\_biosynthetic\_gene\_cluster | 31 | 50.5274847307 | 366 | 1.8e-100 |
| AAB66505.1 | tylactone\_synthase\_module\_3 | BGC0000166 | Tylactone\_biosynthetic\_gene\_cluster | 30 | 56.5796779567 | 366 | 2.4e-100 |
| AJW65407.1 | type\_I\_modular\_polyketide\_synthase | BGC0001195 | Nocardiopsin\_biosynthetic\_gene\_cluster | 31 | 51.3048306496 | 366 | 2.4e-100 |
| AGI99495.1 | Type\_I\_polyketide\_synthase | BGC0001004 | Lobophorin\_biosynthetic\_gene\_cluster | 30 | 50.3053858967 | 365 | 3.1e-100 |
| AEC13070.1 | fosD | BGC0000060 | Fostriecin\_biosynthetic\_gene\_cluster | 31 | 51.1382565242 | 365 | 3.1e-100 |
| ABV83229.1 | CppB | BGC0000116 | Nystatin-like\_Pseudonocardia\_polyene\_biosynthetic\_gene\_cluster | 30 | 49.9722376458 | 365 | 4.1e-100 |
| AJO72737.1 | Type\_I\_modular\_polyketide\_synthase | BGC0001381 | Brasilinolide\_biosynthetic\_gene\_cluster | 31 | 50.8606329817 | 365 | 4.1e-100 |
| BAC20564.1 | polyketide\_synthase | BGC0000039 | Compactin\_biosynthetic\_gene\_cluster | 30 | 53.9700166574 | 364 | 5.3e-100 |
| ABC87511.1 | polyketide\_synthase | BGC0001011 | Meridamycin\_biosynthetic\_gene\_cluster | 31 | 50.4164353137 | 364 | 6.9e-100 |
| ctg1\_orf22 |  | BGC0001013 | Meridamycin\_biosynthetic\_gene\_cluster | 31 | 50.4164353137 | 364 | 6.9e-100 |
| ABJ97438.1 | MerB | BGC0001012 | Meridamycin\_biosynthetic\_gene\_cluster | 30 | 50.5274847307 | 364 | 6.9e-100 |
| ctg1\_orf27 |  | BGC0000096 | Midecamycin\_biosynthetic\_gene\_cluster | 34 | 46.640755136 | 364 | 9.1e-100 |
| AAS79460.1 | polyketide\_synthase\_subunit | BGC0000035 | Chalcomycin\_biosynthetic\_gene\_cluster | 32 | 47.8622987229 | 364 | 9.1e-100 |
| BAO66529.1 | type\_I\_polyketide\_synthase | BGC0000042 | Cremimycin\_biosynthetic\_gene\_cluster | 31 | 51.6935036091 | 364 | 9.1e-100 |
| AAB66506.1 | tylactone\_synthase\_modules\_4\_&\_5 | BGC0000166 | Tylactone\_biosynthetic\_gene\_cluster | 31 | 51.4158800666 | 364 | 9.1e-100 |
| ABJ97437.1 | MerA | BGC0001012 | Meridamycin\_biosynthetic\_gene\_cluster | 30 | 50.6940588562 | 364 | 9.1e-100 |
| AFL48532.1 | laidlomycin\_polyketide\_synthase\_(module\_11\_and\_module\_12) | BGC0000084 | Laidlomycin\_biosynthetic\_gene\_cluster | 30 | 54.9694614103 | 364 | 9.1e-100 |
| CAQ64687.1 | lasalocid\_modular\_polyketide\_synthase | BGC0000087 | Lasalocid\_biosynthetic\_gene\_cluster | 29 | 50.8051082732 | 363 | 1.2e-99 |
| AGC09484.1 | LobS1 | BGC0001183 | Lobophorin\_biosynthetic\_gene\_cluster | 30 | 50.1388117712 | 363 | 1.2e-99 |
| ABA02239.1 | polyketide\_synthase | BGC0000098 | Monacolin\_K\_biosynthetic\_gene\_cluster | 30 | 53.1926707385 | 363 | 1.5e-99 |
| AAO62585.1 | peptide\_sythetase\_polyketide\_synthase\_fusion\_protein | BGC0001016 | Microcystin\_biosynthetic\_gene\_cluster | 30 | 57.1904497501 | 363 | 1.5e-99 |
| AEZ53948.1 | polyketide\_synthase | BGC0000144 | Salinomycin\_biosynthetic\_gene\_cluster | 30 | 53.0816213215 | 363 | 1.5e-99 |
| AAG23264.1 | polyketide\_synthase\_loading\_and\_extender\_module\_1 | BGC0000148 | Spinosad\_biosynthetic\_gene\_cluster | 32 | 50.4164353137 | 363 | 1.5e-99 |
| AAO06917.1 | GdmAII | BGC0000066 | Geldanamycin\_biosynthetic\_gene\_cluster | 30 | 51.4158800666 | 363 | 2e-99 |
| AEZ53946.1 | polyketide\_synthase | BGC0000144 | Salinomycin\_biosynthetic\_gene\_cluster | 30 | 52.0821765686 | 363 | 2e-99 |
| AHB82059.1 | non\_ribosomal\_peptide\_synthetase/polyketide\_synthase | BGC0001019 | Microsclerodermins\_biosynthetic\_gene\_cluster | 32 | 51.4158800666 | 363 | 2e-99 |
| AAM81585.1 | putative\_type\_I\_polyketide\_synthase | BGC0000047 | Dihydrochalcomycin\_biosynthetic\_gene\_cluster | 31 | 47.5846751805 | 362 | 2.6e-99 |
| AAO65806.1 | monensin\_polyketide\_synthase\_modules\_11\_and\_12 | BGC0000100 | Monensin\_biosynthetic\_gene\_cluster | 29 | 51.6935036091 | 362 | 2.6e-99 |
| AAK83194.1 | polyketide\_synthase | BGC0000026 | Avilamycin\_A\_biosynthetic\_gene\_cluster | 30 | 51.2493059411 | 361 | 5.9e-99 |
| AHB82051.1 | polyketide\_synthase | BGC0001019 | Microsclerodermins\_biosynthetic\_gene\_cluster | 32 | 51.5269294836 | 361 | 5.9e-99 |
| AEZ53949.1 | polyketide\_synthase | BGC0000144 | Salinomycin\_biosynthetic\_gene\_cluster | 30 | 52.0266518601 | 361 | 7.7e-99 |
| CAA60462.1 | polyketide\_synthase | BGC0001040 | Rapamycin\_biosynthetic\_gene\_cluster | 30 | 56.1354802887 | 361 | 7.7e-99 |
| CAI94713.1 | putative\_polyketide\_synthase | BGC0000141 | Rubradirin\_biosynthetic\_gene\_cluster | 31 | 50.6940588562 | 360 | 1.3e-98 |
| AAB66504.1 | tylactone\_synthase\_starter\_module\_and\_modules\_1\_&\_2 | BGC0000166 | Tylactone\_biosynthetic\_gene\_cluster | 31 | 51.1382565242 | 360 | 1.3e-98 |
| ACO94500.1 | polyketide\_synthase\_type\_I | BGC0000097 | ML-449\_biosynthetic\_gene\_cluster | 30 | 49.4725152693 | 359 | 1.7e-98 |
| CBZ41586.1 | putative\_modular\_polyketide\_synthase | BGC0000151 | Stambomycin\_biosynthetic\_gene\_cluster | 31 | 50.8051082732 | 359 | 2.2e-98 |
| AEH42473.1 | polyketide\_synthase | BGC0000032 | Calcimycin\_biosynthetic\_gene\_cluster | 31 | 50.1388117712 | 359 | 2.2e-98 |
| ANC94965.1 | AlmHII | BGC0001396 | Aldgamycin\_biosynthetic\_gene\_cluster | 31 | 47.4736257635 | 359 | 2.2e-98 |
| ctg1\_orf524 |  | BGC0001199 | Akaeolide\_biosynthetic\_gene\_cluster | 30 | 49.6946141033 | 359 | 2.2e-98 |
| AHB82065.1 | polyketide\_synthase | BGC0001231 | Microsclerodermins\_biosynthetic\_gene\_cluster | 35 | 38.0344253193 | 359 | 2.9e-98 |
| ABB88520.1 | polyketide\_synthase\_type\_I | BGC0000050 | E-837\_biosynthetic\_gene\_cluster | 31 | 49.9722376458 | 359 | 2.9e-98 |
| CAQ18839.1 | hybrid\_polyketide\_synthase/nonribosomal\_polypetide\_synthetase | BGC0000954 | Ajudazol\_biosynthetic\_gene\_cluster | 32 | 50.7495835647 | 358 | 5e-98 |
| BAF85839.1 | modular\_polyketide\_synthase | BGC0000109 | Nemadectin\_biosynthetic\_gene\_cluster | 29 | 51.0272071072 | 358 | 5e-98 |
| AAM81584.2 | putative\_type\_I\_polyketide\_synthase | BGC0000047 | Dihydrochalcomycin\_biosynthetic\_gene\_cluster | 31 | 50.8051082732 | 358 | 5e-98 |
| IF55\_RS36525 | polyketide\_synthase | BGC0001348 | JBIR-100\_biosynthetic\_gene\_cluster | 30 | 51.1937812326 | 358 | 5e-98 |
| AFL48533.1 | laidlomycin\_polyketide\_synthase\_(module\_10) | BGC0000084 | Laidlomycin\_biosynthetic\_gene\_cluster | 29 | 50.8606329817 | 358 | 5e-98 |
| AFL48525.1 | laidlomycin\_polyketide\_synthase\_(loading\_module\_and\_module\_1) | BGC0000084 | Laidlomycin\_biosynthetic\_gene\_cluster | 31 | 50.7495835647 | 358 | 6.5e-98 |
| ALA09355.1 | type\_I\_modular\_PKS | BGC0001303 | Lobosamide\_biosynthetic\_gene\_cluster | 30 | 49.9722376458 | 358 | 6.5e-98 |
| AEZ53951.1 | polyketide\_synthase | BGC0000144 | Salinomycin\_biosynthetic\_gene\_cluster | 30 | 51.0272071072 | 358 | 6.5e-98 |
| ADU85988.1 | putative\_iterative\_type\_I\_polyketide\_synthase | BGC0000165 | Tiacumicin\_B\_biosynthetic\_gene\_cluster | 30 | 50.6385341477 | 358 | 6.5e-98 |
| AAU93805.2 | polyketide\_synthase\_modules\_5\_and\_6 | BGC0000054 | Erythromycin\_biosynthetic\_gene\_cluster | 30 | 56.5796779567 | 358 | 6.5e-98 |
| ABB88533.1 | polyketide\_synthase\_type\_I | BGC0000049 | E-492\_/\_E-975\_biosynthetic\_gene\_cluster | 31 | 54.6918378679 | 357 | 8.5e-98 |
| ADC45516.1 | modular\_polyketide\_synthase | BGC0000093 | Meilingmycin\_biosynthetic\_gene\_cluster | 31 | 50.4719600222 | 356 | 1.9e-97 |
| ANC94963.1 | AlmHIV | BGC0001396 | Aldgamycin\_biosynthetic\_gene\_cluster | 31 | 50.0277623542 | 355 | 3.2e-97 |
| AHB82072.1 | non\_ribosomal\_peptide\_synthetase/polyketide\_synthase | BGC0001231 | Microsclerodermins\_biosynthetic\_gene\_cluster | 32 | 53.5258189895 | 355 | 3.2e-97 |
| CAO85896.1 | protein\_modular\_polyketide\_synthase\_NorA' | BGC0000110 | Neoaureothin\_biosynthetic\_gene\_cluster | 31 | 51.0272071072 | 355 | 3.2e-97 |
| AAP85336.1 | type\_I\_PKS | BGC0000233 | Hedamycin\_biosynthetic\_gene\_cluster | 31 | 50.6940588562 | 355 | 3.2e-97 |
| ADH01663.1 | putative\_polyketide\_synthase\_PKS3 | BGC0000099 | Monascorubrin\_biosynthetic\_gene\_cluster | 27 | 56.3020544142 | 355 | 4.2e-97 |
| CBA11582.1 | polyketide\_synthase\_type\_I | BGC0001046 | Streptolydigin\_biosynthetic\_gene\_cluster | 30 | 51.1382565242 | 354 | 5.5e-97 |
| AAS79459.1 | polyketide\_synthase\_subunit | BGC0000035 | Chalcomycin\_biosynthetic\_gene\_cluster | 31 | 50.8051082732 | 354 | 5.5e-97 |
| AAF86393.1 | FkbB | BGC0000994 | FK520\_biosynthetic\_gene\_cluster | 30 | 49.9167129373 | 354 | 7.2e-97 |
| AAO65807.1 | monensin\_polyketide\_synthase\_module\_10 | BGC0000100 | Monensin\_biosynthetic\_gene\_cluster | 30 | 50.3609106052 | 354 | 7.2e-97 |
| AGN71604.1 | conidial\_yellow\_pigment\_biosynthesis\_polyketide\_synthase | BGC0000027 | Azaphilone\_biosynthetic\_gene\_cluster | 28 | 56.5241532482 | 354 | 7.2e-97 |
| CUI25672.1 | Thiolactomycin\_Hybrid\_PKS-NRPS\_system | BGC0001353 | Thiolactomycin\_biosynthetic\_gene\_cluster | 30 | 50.1388117712 | 354 | 7.2e-97 |
| ADC79619.1 | BafAIV | BGC0000028 | Bafilomycin\_biosynthetic\_gene\_cluster | 30 | 50.5830094392 | 353 | 1.2e-96 |
| ABB52544.1 | putative\_type\_I\_polyketide\_synthase | BGC0000047 | Dihydrochalcomycin\_biosynthetic\_gene\_cluster | 31 | 50.2498611882 | 353 | 1.2e-96 |
| ACF35447.1 | MbcAIII | BGC0000090 | Macbecin\_biosynthetic\_gene\_cluster | 31 | 50.9716823987 | 352 | 2.1e-96 |
| ABB88519.1 | polyketide\_synthase\_type\_I | BGC0000050 | E-837\_biosynthetic\_gene\_cluster | 31 | 50.4164353137 | 352 | 2.1e-96 |
| AEC13072.1 | fosF | BGC0000060 | Fostriecin\_biosynthetic\_gene\_cluster | 31 | 51.8600777346 | 352 | 2.1e-96 |
| CUW01173.1 | Type\_I\_polyketide\_synthase | BGC0001359 | PM100117\_/\_PM100118\_biosynthetic\_gene\_cluster | 30 | 52.5263742365 | 352 | 2.7e-96 |
| ADC45586.1 | modular\_polyketide\_synthase | BGC0000093 | Meilingmycin\_biosynthetic\_gene\_cluster | 30 | 51.1937812326 | 352 | 2.7e-96 |
| ADA69241.1 | cis-AT\_polyketide\_synthase | BGC0001071 | Nosperin\_biosynthetic\_gene\_cluster | 31 | 50.2498611882 | 352 | 2.7e-96 |
| BAC57030.1 | protomycinolide\_IV\_synthase\_3 | BGC0000102 | Mycinamicin\_biosynthetic\_gene\_cluster | 32 | 51.9711271516 | 352 | 3.6e-96 |
| AAS98784.1 | JamM | BGC0001001 | Jamaicamide\_biosynthetic\_gene\_cluster | 30 | 50.3053858967 | 352 | 3.6e-96 |
| ACO94472.1 | polyketide\_synthase\_type\_I | BGC0000029 | BE-14106\_biosynthetic\_gene\_cluster | 30 | 49.5280399778 | 351 | 4.7e-96 |
| AGY30676.1 | Ann4 | BGC0001298 | 4-Z-annimycin\_biosynthetic\_gene\_cluster | 31 | 50.9716823987 | 351 | 4.7e-96 |
| ACR50795.1 | putative\_polyketide\_synthase | BGC0000163 | Tetronasin\_biosynthetic\_gene\_cluster | 35 | 36.8684064409 | 351 | 4.7e-96 |
| CAD29795.1 | peptide\_synthetase | BGC0001015 | Microcystin\_biosynthetic\_gene\_cluster | 30 | 55.0805108273 | 351 | 6.1e-96 |
| AJO72743.1 | Type\_I\_modular\_polyketide\_synthase | BGC0001381 | Brasilinolide\_biosynthetic\_gene\_cluster | 32 | 50.5274847307 | 351 | 6.1e-96 |
| AAX98190.1 | polyketide\_synthase\_type\_I | BGC0000052 | ECO-02301\_biosynthetic\_gene\_cluster | 30 | 50.5274847307 | 351 | 7.9e-96 |
| AEZ53952.1 | polyketide\_synthase | BGC0000144 | Salinomycin\_biosynthetic\_gene\_cluster | 29 | 50.6385341477 | 351 | 7.9e-96 |
| ADB12490.1 | EpoC | BGC0000990 | Epothilone\_biosynthetic\_gene\_cluster | 30 | 50.5830094392 | 351 | 7.9e-96 |
| AAV66110.2 | fusaridione\_A\_synthetase | BGC0000992 | Fusaridione\_A\_biosynthetic\_gene\_cluster | 31 | 47.5846751805 | 350 | 1e-95 |
| AAF26920.1 | polyketide\_synthase | BGC0000988 | Epothilone\_biosynthetic\_gene\_cluster | 30 | 50.5830094392 | 350 | 1.4e-95 |
| AJO72735.1 | Type\_I\_modular\_polyketide\_synthase | BGC0001381 | Brasilinolide\_biosynthetic\_gene\_cluster | 30 | 52.5263742365 | 350 | 1.4e-95 |
| BAE93731.1 | type\_I\_polyketide\_synthase | BGC0000164 | Tetronomycin\_biosynthetic\_gene\_cluster | 31 | 46.2520821766 | 349 | 2.3e-95 |
| ABP55210.1 | beta-ketoacyl\_synthase | BGC0000142 | Salinilactam\_biosynthetic\_gene\_cluster | 30 | 51.9156024431 | 349 | 2.3e-95 |
| BAC57031.1 | protomycinolide\_IV\_synthase\_4 | BGC0000102 | Mycinamicin\_biosynthetic\_gene\_cluster | 32 | 51.7490283176 | 349 | 3e-95 |
| CAQ34918.1 | nonribosomal\_peptide\_synthetase/\_polyketide\_synthase | BGC0000986 | Dkxanthene\_biosynthetic\_gene\_cluster | 31 | 49.3614658523 | 348 | 5.1e-95 |
| AAQ84146.1 | Plm6 | BGC0000123 | Phoslactomycin\_B\_biosynthetic\_gene\_cluster | 29 | 49.5835646863 | 347 | 6.7e-95 |
| AAF62882.1 | EpoC | BGC0000991 | Epothilone\_biosynthetic\_gene\_cluster | 30 | 50.5830094392 | 347 | 8.8e-95 |
| AAD03048.1 | type\_I\_polyketide\_synthase | BGC0000041 | Coronafacic\_acid\_biosynthetic\_gene\_cluster | 29 | 51.0272071072 | 347 | 1.1e-94 |
| AAP42860.1 | NanA6 | BGC0000105 | Nanchangmycin\_biosynthetic\_gene\_cluster | 30 | 51.2493059411 | 346 | 2e-94 |
| ADZ24995.1 | non-ribosomal\_peptide\_synthase/polyketide\_synthase | BGC0000380 | Leupyrrin\_biosynthetic\_gene\_cluster | 30 | 55.6357579123 | 346 | 2.6e-94 |
| BAE93728.1 | type\_I\_polyketide\_synthase | BGC0000164 | Tetronomycin\_biosynthetic\_gene\_cluster | 30 | 50.3053858967 | 345 | 3.3e-94 |
| ADA82585.1 | hybrid\_trans-AT\_polyketide\_synthase\_-\_nonribosomal\_peptide\_synthetase | BGC0001110 | Psymberin\_biosynthetic\_gene\_cluster | 35 | 35.646862854 | 345 | 4.4e-94 |
| AHV78252.1 | ResS1 | BGC0001246 | Trans-resorcylide\_biosynthetic\_gene\_cluster | 30 | 51.7490283176 | 344 | 5.7e-94 |
| CBZ41585.1 | putative\_modular\_polyketide\_synthase | BGC0000151 | Stambomycin\_biosynthetic\_gene\_cluster | 30 | 50.5830094392 | 344 | 5.7e-94 |
| AAY89049.1 | polyketide\_synthase | BGC0001069 | Chivosazole\_biosynthetic\_gene\_cluster | 30 | 48.8062187674 | 344 | 9.7e-94 |
| BI06\_RS39060 | polyketide\_synthase | BGC0001232 | Leucanicidin\_biosynthetic\_gene\_cluster | 29 | 52.0821765686 | 343 | 1.7e-93 |
| BAK26562.1 | PKS-NRPS\_hybrid | BGC0000977 | Cyclopiazonic\_acid\_biosynthetic\_gene\_cluster | 29 | 51.9156024431 | 342 | 2.8e-93 |
| AAF15892.2 | NosB | BGC0001028 | Nostopeptolide\_biosynthetic\_gene\_cluster | 30 | 50.5830094392 | 342 | 3.7e-93 |
| AEZ53950.1 | polyketide\_synthase | BGC0000144 | Salinomycin\_biosynthetic\_gene\_cluster | 29 | 51.3603553581 | 341 | 4.8e-93 |
| ADC79616.1 | BafAI | BGC0000028 | Bafilomycin\_biosynthetic\_gene\_cluster | 29 | 50.5274847307 | 341 | 4.8e-93 |
| BAF85838.1 | modular\_polyketide\_synthase | BGC0000109 | Nemadectin\_biosynthetic\_gene\_cluster | 28 | 51.7490283176 | 341 | 6.3e-93 |
| BAC20566.1 | polyketide\_synthase | BGC0000039 | Compactin\_biosynthetic\_gene\_cluster | 29 | 52.692948362 | 340 | 1.1e-92 |
| CAD89775.1 | MelD\_protein | BGC0001009 | Melithiazol\_biosynthetic\_gene\_cluster | 29 | 54.6363131594 | 340 | 1.1e-92 |
| AAY28225.1 | HbmAI | BGC0000074 | Herbimycin\_biosynthetic\_gene\_cluster | 29 | 53.13714603 | 340 | 1.1e-92 |
| AAS98200.1 | MSAS-type\_polyketide\_synthase | BGC0001273 | Asperlactone\_biosynthetic\_gene\_cluster | 30 | 48.6951693504 | 340 | 1.4e-92 |
| ABY21540.1 | AngAIII | BGC0000018 | Angolamycin\_biosynthetic\_gene\_cluster | 30 | 51.9156024431 | 340 | 1.4e-92 |
| EWG54266.1 | hypothetical\_protein | BGC0001190 | Fusaric\_acid\_biosynthetic\_gene\_cluster | 29 | 53.13714603 | 340 | 1.4e-92 |
| AJO72734.1 | Type\_I\_modular\_polyketide\_synthase | BGC0001381 | Brasilinolide\_biosynthetic\_gene\_cluster | 30 | 50.2498611882 | 339 | 1.8e-92 |
| AAF86392.1 | FkbC | BGC0000994 | FK520\_biosynthetic\_gene\_cluster | 29 | 50.3609106052 | 339 | 2.4e-92 |
| AAW03327.1 | CtaD | BGC0000982 | Cystothiazole\_A\_biosynthetic\_gene\_cluster | 29 | 54.5807884509 | 338 | 4.1e-92 |
| BAQ25466.1 | polyketide\_synthase | BGC0001280 | Betaenone\_C\_/\_betaenone\_A\_biosynthetic\_gene\_cluster | 30 | 52.4153248195 | 337 | 1.2e-91 |
| BAE93725.1 | type\_I\_polyketide\_synthase | BGC0000164 | Tetronomycin\_biosynthetic\_gene\_cluster | 30 | 51.3048306496 | 337 | 1.2e-91 |
| AAC68815.1 | FK506\_polyketide\_synthase | BGC0000353 | FK506\_biosynthetic\_gene\_cluster | 30 | 50.2498611882 | 337 | 1.2e-91 |
| AAS79462.1 | polyketide\_synthase\_subunit | BGC0000035 | Chalcomycin\_biosynthetic\_gene\_cluster | 31 | 50.4719600222 | 336 | 2e-91 |
| BAK64650.1 | polyketide\_synthase | BGC0000135 | Reveromycin\_biosynthetic\_gene\_cluster | 30 | 50.9716823987 | 336 | 2e-91 |
| AHV78245.1 | LasS1 | BGC0001245 | Lasiodiplodin\_biosynthetic\_gene\_cluster | 30 | 52.1377012771 | 335 | 3.5e-91 |
| AGO86662.1 | equisetin\_synthetase | BGC0001255 | Equisetin\_biosynthetic\_gene\_cluster | 29 | 47.196002221 | 335 | 4.5e-91 |
| BAO98805.1 | putative\_polyketide\_synthase | BGC0001002 | Jawsamycin\_biosynthetic\_gene\_cluster | 30 | 50.4164353137 | 333 | 1.3e-90 |
| CAF05649.1 | TubD\_protein | BGC0001053 | Tubulysin\_biosynthetic\_gene\_cluster | 31 | 51.3048306496 | 332 | 2.9e-90 |
| ALJ49921.1 | TtmH | BGC0001236 | Thiotetroamide\_biosynthetic\_gene\_cluster | 28 | 56.5796779567 | 331 | 6.5e-90 |
| ACD39767.1 | reducing\_polyketide\_synthase | BGC0000077 | Hypothemycin\_biosynthetic\_gene\_cluster | 30 | 50.9716823987 | 331 | 6.5e-90 |
| ACD39758.1 | reducing\_polyketide\_synthase | BGC0000076 | Hypothemycin\_biosynthetic\_gene\_cluster | 30 | 50.9716823987 | 331 | 6.5e-90 |
| AAQ84144.1 | Plm4 | BGC0000123 | Phoslactomycin\_B\_biosynthetic\_gene\_cluster | 30 | 48.4730705164 | 331 | 8.5e-90 |
| ABB90283.1 | polyketide\_synthase | BGC0001057 | Zearalenone\_biosynthetic\_gene\_cluster | 30 | 50.8051082732 | 330 | 1.1e-89 |
| ABL74938.1 | TlmVIII | BGC0001048 | Tallysomycin\_biosynthetic\_gene\_cluster | 31 | 50.5830094392 | 330 | 1.5e-89 |
| CAF05651.1 | TubF\_protein | BGC0001053 | Tubulysin\_biosynthetic\_gene\_cluster | 32 | 50.6940588562 | 329 | 2.5e-89 |
| BAE93739.1 | type\_I\_polyketide\_synthase-related\_protein | BGC0000164 | Tetronomycin\_biosynthetic\_gene\_cluster | 34 | 37.6457523598 | 328 | 4.2e-89 |
| ABA02240.1 | polyketide\_synthase | BGC0000098 | Monacolin\_K\_biosynthetic\_gene\_cluster | 29 | 52.2487506941 | 328 | 5.5e-89 |
| CAO98848.1 | polyketide\_synthase\_AufE | BGC0000023 | Aurafuron\_biosynthetic\_gene\_cluster | 34 | 38.7007218212 | 327 | 7.2e-89 |
| AAD34559.1 | polyketide\_synthase | BGC0000089 | Lovastatin\_biosynthetic\_gene\_cluster | 31 | 43.0316490838 | 327 | 1.2e-88 |
| EAQ86385.1 | hypothetical\_protein | BGC0001405 | Chaetoviridin\_/\_chaetomugilin\_biosynthetic\_gene\_cluster | 29 | 44.4197667962 | 327 | 1.2e-88 |
| ABO15861.1 | polyketide\_synthase | BGC0000130 | Pyrrolomycin\_biosynthetic\_gene\_cluster | 35 | 37.4791782343 | 325 | 3.6e-88 |
| AKC54422.1 | fumosorinone\_biosynthesis\_polyketide\_synthase | BGC0001218 | Fumosorinone\_biosynthetic\_gene\_cluster | 30 | 49.3059411438 | 324 | 6.1e-88 |
| AAF00957.1 | McyG | BGC0001017 | Microcystin\_biosynthetic\_gene\_cluster | 29 | 51.7490283176 | 324 | 6.1e-88 |
| EAU29529.1 | hypothetical\_protein | BGC0000682 | Terretonin\_biosynthetic\_gene\_cluster | 37 | 30.4830649639 | 324 | 8e-88 |
| AFD30954.1 | CrmA | BGC0000966 | Caerulomycin\_A\_biosynthetic\_gene\_cluster | 31 | 50.7495835647 | 324 | 1e-87 |
| AGC95324.1 | CurS1 | BGC0000045 | Dehydrocurvularin\_biosynthetic\_gene\_cluster | 29 | 51.9156024431 | 323 | 1.8e-87 |
| CBF87072.1 | polyketide\_synthase,\_putative\_(Eurofung) | BGC0001290 | Emericellamide\_A\_/\_emericellamide\_B\_biosynthetic\_gene\_cluster | 29 | 52.3042754026 | 323 | 1.8e-87 |
| CAA73127.1 | HMWP1\_protein | BGC0000467 | Yersiniabactin\_biosynthetic\_gene\_cluster | 28 | 49.6390893948 | 323 | 1.8e-87 |
| BAD97694.1 | Aft9-1 | BGC0000003 | AF-toxin\_biosynthetic\_gene\_cluster | 31 | 45.9744586341 | 322 | 3e-87 |
| CUI25702.1 | Tu3010\_Hybrid\_PKS-NRPS\_system | BGC0001352 | Thiotetronate\_Tu\_3010\_biosynthetic\_gene\_cluster | 28 | 56.4686285397 | 322 | 4e-87 |
| AMY15068.1 | hexaketide\_synthase\_MF-SQHKS | BGC0001339 | Squalestatin\_S1\_biosynthetic\_gene\_cluster | 28 | 50.8051082732 | 320 | 1.5e-86 |
| ADI59531.1 | CorI | BGC0001091 | Corallopyronin\_biosynthetic\_gene\_cluster | 37 | 31.4825097168 | 319 | 3.3e-86 |
| CAJ87591.1 | putative\_peptide/polyketide\_synthetase | BGC0001055 | Yersiniabactin\_biosynthetic\_gene\_cluster | 28 | 49.6390893948 | 318 | 5.7e-86 |
| AGC09486.1 | LobS3 | BGC0001183 | Lobophorin\_biosynthetic\_gene\_cluster | 33 | 33.6479733481 | 318 | 5.7e-86 |
| EHA19289.1 | hypothetical\_protein | BGC0001124 | Pyranonigrin\_E\_biosynthetic\_gene\_cluster | 29 | 51.7490283176 | 318 | 5.7e-86 |
| CAL80821.1 | sylD-like\_NRPS/PKS | BGC0000997 | Glidobactin\_biosynthetic\_gene\_cluster | 30 | 51.5269294836 | 318 | 5.7e-86 |
| CUI25727.1 | Polyketide\_synthase/Nonribosomal\_peptide\_synthase | BGC0001355 | U-68204\_biosynthetic\_gene\_cluster | 29 | 51.0827318157 | 317 | 9.7e-86 |
| EAU38971.1 | hypothetical\_protein | BGC0001122 | Isoflavipucine\_biosynthetic\_gene\_cluster | 31 | 48.4730705164 | 317 | 1.3e-85 |
| AGY62753.1 | EbeA | BGC0000051 | Ebelactone\_biosynthetic\_gene\_cluster | 31 | 45.9744586341 | 316 | 1.7e-85 |
| CAQ34917.1 | polyketide\_synthase | BGC0000986 | Dkxanthene\_biosynthetic\_gene\_cluster | 34 | 36.9794558579 | 316 | 1.7e-85 |
| AKL78824.1 | GLPKS3 | BGC0001187 | Xenolozoyenone\_biosynthetic\_gene\_cluster | 28 | 52.5263742365 | 316 | 2.8e-85 |
| EAW09117.1 | hybrid\_NRPS/PKS\_enzyme,\_putative | BGC0000983 | Cytochalasin\_biosynthetic\_gene\_cluster | 29 | 51.6379789006 | 315 | 3.7e-85 |
| CCM44338.1 | putative\_polyketide\_synthase | BGC0001056 | Zeamine\_biosynthetic\_gene\_cluster | 29 | 51.0272071072 | 315 | 3.7e-85 |
| ABP73645.1 | SalA | BGC0000145 | Salinosporamide\_A\_biosynthetic\_gene\_cluster | 30 | 48.8062187674 | 315 | 4.8e-85 |
| ACD39774.1 | reducing\_polyketide\_synthase | BGC0000134 | Radicicol\_biosynthetic\_gene\_cluster | 29 | 51.6379789006 | 315 | 4.8e-85 |
| AHB82070.1 | polyketide\_synthase | BGC0001231 | Microsclerodermins\_biosynthetic\_gene\_cluster | 29 | 51.0827318157 | 314 | 8.2e-85 |
| ABF87031.1 | non-ribosomal\_peptide\_synthetase/polyketide\_synthase | BGC0000393 | Myxoprincomide\_biosynthetic\_gene\_cluster | 29 | 50.8606329817 | 314 | 8.2e-85 |
| ABX37384.1 | Beta-ketoacyl\_synthase | BGC0000984 | Delftibactin\_biosynthetic\_gene\_cluster | 30 | 51.8045530261 | 314 | 1.1e-84 |
| ABP53498.1 | beta-ketoacyl\_synthase | BGC0001041 | Salinosporamide\_biosynthetic\_gene\_cluster | 30 | 48.8062187674 | 313 | 1.4e-84 |
| AGZ15472.1 | putative\_modular\_polyketide\_synthase | BGC0001036 | Polyoxypeptin\_biosynthetic\_gene\_cluster | 30 | 52.3042754026 | 312 | 2.4e-84 |
| ACZ57548.1 | polyketide\_synthase | BGC0000046 | Depudecin\_biosynthetic\_gene\_cluster | 28 | 52.803997779 | 312 | 3.1e-84 |
| EHA22196.1 | polyketide\_synthase | BGC0000170 | Yanuthone\_D\_biosynthetic\_gene\_cluster | 32 | 41.8101054969 | 309 | 2.6e-83 |
| CAD70195.1 | non-ribosomal\_peptide\_synthetase | BGC0001047 | Syringolin\_A\_biosynthetic\_gene\_cluster | 29 | 52.4153248195 | 309 | 3.5e-83 |
| AAG02357.1 | polyketide\_synthase | BGC0000963 | Bleomycin\_biosynthetic\_gene\_cluster | 30 | 49.8611882288 | 308 | 4.5e-83 |
| ADH04682.1 | polyketide\_synthase | BGC0001344 | Tubulysin\_biosynthetic\_gene\_cluster | 30 | 50.8051082732 | 305 | 2.9e-82 |
| KGO40478.1 | Acyl\_transferase/acyl\_hydrolase/lysophospholipase | BGC0001205 | Communesin\_biosynthetic\_gene\_cluster | 29 | 52.4153248195 | 304 | 1.1e-81 |
| CCC55921.1 | non-ribosomal\_peptide\_synthetase/polyketide\_synthase\_hybrid\_protein | BGC0000973 | Collismycin\_A\_biosynthetic\_gene\_cluster | 30 | 52.1377012771 | 300 | 1.6e-80 |
| CAJ76298.1 | putative\_hybrid\_polyketide-non-ribosomal\_peptide\_synthetase | BGC0000972 | Colibactin\_biosynthetic\_gene\_cluster | 29 | 50.3053858967 | 300 | 1.6e-80 |
| ABB76806.1 | polyketide\_synthase\_2 | BGC0000155\_c2 | T-toxin\_biosynthetic\_gene\_cluster | 27 | 51.1382565242 | 299 | 2.1e-80 |
| BAD83684.1 | PKSN\_polyketide\_synthase\_for\_alternapyrone\_biosynthesis | BGC0000012 | Alternapyrone\_biosynthetic\_gene\_cluster | 37 | 25.7079400333 | 298 | 6.1e-80 |
| AHB82057.1 | polyketide\_synthase | BGC0001019 | Microsclerodermins\_biosynthetic\_gene\_cluster | 29 | 52.8595224875 | 297 | 1.4e-79 |
| AIW00670.1 | mellein\_synthase | BGC0001244 | (-)-Mellein\_biosynthetic\_gene\_cluster | 27 | 50.0277623542 | 296 | 2.3e-79 |
| CAE14173.1 |  | BGC0000383 | Luminmycin\_biosynthetic\_gene\_cluster | 29 | 50.3053858967 | 295 | 3e-79 |
| AEZ54376.1 | PieA3 | BGC0000124 | Piericidin\_A1\_biosynthetic\_gene\_cluster | 32 | 39.0893947807 | 294 | 6.8e-79 |
| CAN93347.1 | Polyketide\_synthase | BGC0000179 | Etnangien\_biosynthetic\_gene\_cluster | 37 | 29.483620211 | 293 | 2e-78 |
| BAP05593.1 | CalE | BGC0000967 | Calyculin\_biosynthetic\_gene\_cluster | 37 | 29.039422543 | 292 | 2.6e-78 |
| CAN93348.1 | polyketide\_synthase | BGC0000179 | Etnangien\_biosynthetic\_gene\_cluster | 36 | 28.928373126 | 292 | 2.6e-78 |
| EAA65604.1 | hypothetical\_protein | BGC0000022 | Asperfuranone\_biosynthetic\_gene\_cluster | 26 | 53.6923931149 | 291 | 5.7e-78 |
| AHE80993.1 | PieA3 | BGC0001169 | Piericidin\_A1\_biosynthetic\_gene\_cluster | 32 | 37.3681288173 | 289 | 2.2e-77 |
| AEC13071.1 | fosE | BGC0000060 | Fostriecin\_biosynthetic\_gene\_cluster | 38 | 25.0971682399 | 289 | 2.2e-77 |
| AFN27480.1 | pks\_BonA | BGC0000173 | Bongkrekic\_acid\_biosynthetic\_gene\_cluster | 37 | 28.9838978345 | 289 | 2.8e-77 |
| ALD83686.1 | tAT\_polyketide\_synthase | BGC0001300 | Anthracimycin\_biosynthetic\_gene\_cluster | 38 | 26.4297612438 | 289 | 3.7e-77 |
| AIU36103.1 | LglD | BGC0000180 | Legioliulin\_biosynthetic\_gene\_cluster | 35 | 29.5391449195 | 289 | 3.7e-77 |
| CAJ57411.1 | polyketide\_synthase\_type\_I | BGC0000176 | Difficidin\_biosynthetic\_gene\_cluster | 35 | 31.3159355913 | 288 | 6.3e-77 |
| AGC09485.1 | LobS2 | BGC0001183 | Lobophorin\_biosynthetic\_gene\_cluster | 36 | 25.7079400333 | 287 | 1.1e-76 |
| ALD83703.1 | tAT\_polyketide\_synthase | BGC0001299 | Chlorotonil\_biosynthetic\_gene\_cluster | 37 | 30.2609661299 | 287 | 1.1e-76 |
| AEC04361.1 | polyketide\_synthase | BGC0000178 | Elansolid\_biosynthetic\_gene\_cluster | 35 | 28.0399777901 | 287 | 1.1e-76 |
| EPE34340.1 | Thiolase-like\_protein | BGC0001035 | Pneumocandin\_biosynthetic\_gene\_cluster | 36 | 24.8195446974 | 286 | 1.8e-76 |
| AFB35635.1 | EsmB4 | BGC0000935 | Esmeraldin\_biosynthetic\_gene\_cluster | 33 | 39.6446418656 | 286 | 1.8e-76 |
| BAK64638.1 | polyketide\_synthase | BGC0000135 | Reveromycin\_biosynthetic\_gene\_cluster | 37 | 24.3198223209 | 285 | 4.1e-76 |
| CAL69894.1 | RhiF\_protein | BGC0001112 | Rhizoxins\_biosynthetic\_gene\_cluster | 37 | 28.5397001666 | 285 | 4.1e-76 |
| ACY01391.1 | AT-less\_polyketide\_synthase | BGC0000177 | Dorrigocin\_/\_migrastatin\_biosynthetic\_gene\_cluster | 36 | 29.5391449195 | 285 | 5.4e-76 |
| ADC79618.1 | BafAIII | BGC0000028 | Bafilomycin\_biosynthetic\_gene\_cluster | 37 | 24.7640199889 | 285 | 5.4e-76 |
| ALD83688.1 | tAT\_polyketide\_synthase | BGC0001300 | Anthracimycin\_biosynthetic\_gene\_cluster | 32 | 31.8156579678 | 284 | 7e-76 |
| AFX60336.1 | polyketide\_synthase | BGC0001032 | Oocydin\_A\_biosynthetic\_gene\_cluster | 35 | 29.6501943365 | 284 | 7e-76 |
| ABI91465.1 | beta-ketoacyl\_synthase | BGC0001094 | Enacyloxin\_biosynthetic\_gene\_cluster | 33 | 35.9800111049 | 284 | 9.1e-76 |
| ABC35522.1 | thiotemplate\_mechanism\_natural\_product\_synthetase | BGC0000186 | Thailandamide\_biosynthetic\_gene\_cluster | 35 | 30.3164908384 | 284 | 9.1e-76 |
| ABC34675.1 | polyketide\_synthase,\_putative | BGC0000186 | Thailandamide\_biosynthetic\_gene\_cluster | 35 | 30.3720155469 | 284 | 9.1e-76 |
| ADN68483.1 | SorH | BGC0000184 | Sorangicin\_biosynthetic\_gene\_cluster | 36 | 29.261521377 | 284 | 9.1e-76 |
| ACR13065.1 | modular\_polyketide\_synthase,\_type\_I\_PKS | BGC0000185 | Tartrolon\_biosynthetic\_gene\_cluster | 32 | 34.0921710161 | 284 | 1.2e-75 |
| ADN68476.1 | SorA | BGC0000184 | Sorangicin\_biosynthetic\_gene\_cluster | 35 | 29.705719045 | 284 | 1.2e-75 |
| ADN68480.1 | SorE | BGC0000184 | Sorangicin\_biosynthetic\_gene\_cluster | 36 | 30.7051637979 | 284 | 1.2e-75 |
| AFN27482.1 | pks\_BonC | BGC0000173 | Bongkrekic\_acid\_biosynthetic\_gene\_cluster | 35 | 31.0383120489 | 283 | 1.6e-75 |
| AKA59448.1 | polyketide\_synthase | BGC0001203 | Clarexpoxcin\_biosynthetic\_gene\_cluster | 33 | 43.0871737923 | 283 | 2e-75 |
| ALD82523.1 | polyketide\_synthase | BGC0001212 | Nannocystin\_biosynthetic\_gene\_cluster | 40 | 24.2087729039 | 282 | 2.7e-75 |
| ADI59532.1 | CorJ | BGC0001091 | Corallopyronin\_biosynthetic\_gene\_cluster | 37 | 27.2626318712 | 282 | 4.5e-75 |
| ACY01401.1 | AT-less\_polyketide\_synthase | BGC0000083 | Lactimidomycin\_biosynthetic\_gene\_cluster | 36 | 29.816768462 | 282 | 4.5e-75 |
| ABC34832.1 | polyketide\_synthase | BGC0000186 | Thailandamide\_biosynthetic\_gene\_cluster | 35 | 29.0949472515 | 282 | 4.5e-75 |
| CAG23967.1 | polyketide\_synthase\_type\_I | BGC0000181 | Macrolactin\_biosynthetic\_gene\_cluster | 32 | 34.8139922265 | 281 | 5.9e-75 |
| AAP33839.2 | ochratoxin\_A\_polyketide\_synthase | BGC0001030 | Ochratoxin\_A\_biosynthetic\_gene\_cluster | 28 | 41.6435313715 | 281 | 5.9e-75 |
| AIJ04684.1 | polyketide\_synthase | BGC0001383 | Macrolactin\_biosynthetic\_gene\_cluster | 32 | 34.758467518 | 281 | 7.7e-75 |
| ABS90475.1 | PKS | BGC0001106 | Oxazolomycin\_biosynthetic\_gene\_cluster | 37 | 28.6507495836 | 280 | 1e-74 |
| CTQ34881.1 | AtcD;\_polyketide\_synthase,\_modules\_1-4 | BGC0001301 | Anthracimycin\_biosynthetic\_gene\_cluster | 36 | 26.7073847862 | 280 | 1.3e-74 |
| AAY32964.1 | DszA | BGC0001093 | Disorazole\_biosynthetic\_gene\_cluster | 37 | 28.3176013326 | 280 | 1.3e-74 |
| CBX87032.1 | BcBOA9\_protein | BGC0001243\_c3 | Botryenalol\_/\_botrydial\_/\_dihydrobotrydial\_/\_botryendial\_/ | 27 | 53.0816213215 | 280 | 1.3e-74 |
| ALD83687.1 | tAT\_polyketide\_synthase | BGC0001300 | Anthracimycin\_biosynthetic\_gene\_cluster | 37 | 26.5963353692 | 280 | 1.3e-74 |
| ADD82940.1 | Bat2 | BGC0001099 | Kalimantacin\_/\_batumin\_biosynthetic\_gene\_cluster | 35 | 29.7612437535 | 280 | 1.3e-74 |
| ACJ24875.1 | 6-methylsalicylic\_acid\_synthase | BGC0000119 | Pactamycin\_biosynthetic\_gene\_cluster | 40 | 25.3192670738 | 279 | 2.2e-74 |
| BAF92601.1 | iterative\_type\_I\_PKS | BGC0000118 | Pactamycin\_biosynthetic\_gene\_cluster | 40 | 25.3192670738 | 279 | 2.2e-74 |
| CAG23958.2 | polyketide\_synthase | BGC0001089 | Bacillaene\_biosynthetic\_gene\_cluster | 35 | 30.2054414214 | 278 | 6.5e-74 |
| AAM12909.2 | MmpA | BGC0000182 | Mupirocin\_biosynthetic\_gene\_cluster | 35 | 29.3170460855 | 278 | 6.5e-74 |
| CAG23957.2 | NRPS/PKS\_protein | BGC0001089 | Bacillaene\_biosynthetic\_gene\_cluster | 35 | 29.8722931705 | 278 | 6.5e-74 |
| AMH40443.1 | PKS | BGC0001350 | Phormidolide\_biosynthetic\_gene\_cluster | 35 | 28.2620766241 | 277 | 1.5e-73 |
| CBK62724.1 |  | BGC0001115 | Thiomarinol\_biosynthetic\_gene\_cluster | 35 | 29.6501943365 | 277 | 1.5e-73 |
| AFO59866.1 | ChxE | BGC0000175 | Cycloheximide\_/\_actiphenol\_biosynthetic\_gene\_cluster | 35 | 29.483620211 | 276 | 1.9e-73 |
| ABF85931.1 | non-ribosomal\_peptide\_synthase/polyketide\_synthase\_Ta1 | BGC0001025 | Myxovirescin\_biosynthetic\_gene\_cluster | 36 | 29.7612437535 | 276 | 1.9e-73 |
| ABC36203.1 | thiotemplate\_mechanism\_natural\_product\_synthetase | BGC0001102 | Malleilactone\_biosynthetic\_gene\_cluster | 30 | 53.6368684064 | 276 | 2.5e-73 |
| AHA38200.1 | GphG | BGC0000069 | Gephyronic\_acid\_biosynthetic\_gene\_cluster | 33 | 37.4236535258 | 276 | 2.5e-73 |
| AFX60313.1 | polyketide\_synthase | BGC0001031 | Oocydin\_A\_biosynthetic\_gene\_cluster | 35 | 29.927817879 | 275 | 5.5e-73 |
| AAY32965.1 | DszB | BGC0001093 | Disorazole\_biosynthetic\_gene\_cluster | 37 | 29.7612437535 | 275 | 5.5e-73 |
| AEC04364.1 | polyketide\_synthase | BGC0000178 | Elansolid\_biosynthetic\_gene\_cluster | 36 | 29.594669628 | 274 | 9.4e-73 |
| CBJ89760.1 | Polyketide\_synthase\_involved\_in\_xenocoumacin\_synthesis | BGC0001054 | Xenocoumacin\_biosynthetic\_gene\_cluster | 29 | 50.3609106052 | 274 | 9.4e-73 |
| AAN85523.1 | polyketide\_synthase | BGC0001101 | Leinamycin\_biosynthetic\_gene\_cluster | 35 | 31.8156579678 | 274 | 9.4e-73 |
| ADI59533.1 | CorK | BGC0001091 | Corallopyronin\_biosynthetic\_gene\_cluster | 34 | 27.7623542476 | 274 | 1.2e-72 |
| CAN93350.1 | polyketide\_synthase | BGC0000179 | Etnangien\_biosynthetic\_gene\_cluster | 34 | 32.3153803443 | 274 | 1.2e-72 |
| ADA69239.2 | trans-AT\_hybrid\_polyketide\_synthase-NRPS | BGC0001071 | Nosperin\_biosynthetic\_gene\_cluster | 35 | 29.3170460855 | 274 | 1.2e-72 |
| ALD83704.1 | tAT\_polyketide\_synthase | BGC0001299 | Chlorotonil\_biosynthetic\_gene\_cluster | 35 | 30.8162132149 | 273 | 1.6e-72 |
| BAP05595.1 | CalG | BGC0000967 | Calyculin\_biosynthetic\_gene\_cluster | 36 | 27.0960577457 | 273 | 1.6e-72 |
| BAP05594.1 | CalF | BGC0000967 | Calyculin\_biosynthetic\_gene\_cluster | 36 | 28.4286507496 | 273 | 1.6e-72 |
| CBK62731.1 |  | BGC0001115 | Thiomarinol\_biosynthetic\_gene\_cluster | 33 | 30.4275402554 | 273 | 2.1e-72 |
| ABY21539.1 | AngAII | BGC0000018 | Angolamycin\_biosynthetic\_gene\_cluster | 36 | 25.5413659078 | 272 | 2.7e-72 |
| CCP20050.1 | divL3\_protein | BGC0001119 | Divergolide\_biosynthetic\_gene\_cluster | 32 | 28.7617990006 | 272 | 3.6e-72 |
| ABF89568.1 | polyketide\_synthase | BGC0001025 | Myxovirescin\_biosynthetic\_gene\_cluster | 34 | 28.5397001666 | 272 | 4.7e-72 |
| CBJ89764.1 | Polyketide\_synthase\_involved\_in\_xenocoumacin\_synthesis | BGC0001054 | Xenocoumacin\_biosynthetic\_gene\_cluster | 27 | 51.2493059411 | 272 | 4.7e-72 |
| ABS90471.1 | PKS\_type\_I | BGC0001106 | Oxazolomycin\_biosynthetic\_gene\_cluster | 34 | 28.8728484175 | 271 | 8e-72 |
| ABM63527.1 | BryB | BGC0000174 | Bryostatin\_biosynthetic\_gene\_cluster | 34 | 29.8722931705 | 271 | 8e-72 |
| AEC04363.1 | polyketide\_synthase | BGC0000178 | Elansolid\_biosynthetic\_gene\_cluster | 34 | 29.372570794 | 270 | 1.4e-71 |
| AFX60311.1 | polyketide\_synthase | BGC0001031 | Oocydin\_A\_biosynthetic\_gene\_cluster | 34 | 29.5391449195 | 270 | 1.4e-71 |
| AKQ22696.1 | malonyl\_CoA-acyl\_carrier\_protein\_transacylase | BGC0001186 | Misakinolide\_biosynthetic\_gene\_cluster | 35 | 29.705719045 | 270 | 1.8e-71 |
| AFN27483.1 | pks\_BonD | BGC0000173 | Bongkrekic\_acid\_biosynthetic\_gene\_cluster | 35 | 28.928373126 | 270 | 1.8e-71 |
| AMY15057.1 | tetraketide\_synthase\_MF-SQTKS | BGC0001339 | Squalestatin\_S1\_biosynthetic\_gene\_cluster | 34 | 25.7079400333 | 269 | 2.3e-71 |
| BAF50727.1 | hybrid\_polyketide\_synthase-non\_ribosomal\_peptide\_synthetase | BGC0001116 | Virginiamycin\_biosynthetic\_gene\_cluster | 37 | 25.8745141588 | 269 | 3e-71 |
| BAP34739.1 | type\_I\_polyketide\_synthase | BGC0000078 | Incednine\_biosynthetic\_gene\_cluster | 37 | 25.5968906163 | 269 | 3e-71 |
| CAJ57409.1 | polyketide\_synthase\_type\_I | BGC0000176 | Difficidin\_biosynthetic\_gene\_cluster | 34 | 30.9827873404 | 269 | 3e-71 |
| CBK62733.1 |  | BGC0001115 | Thiomarinol\_biosynthetic\_gene\_cluster | 35 | 27.5957801222 | 269 | 4e-71 |
| ADA82581.1 | trans-AT\_polyketide\_synthase | BGC0001110 | Psymberin\_biosynthetic\_gene\_cluster | 37 | 27.9289283731 | 269 | 4e-71 |
| BAC76471.1 | type\_I\_polyketide\_synthase\_LkcF | BGC0001100 | Lankacidin\_biosynthetic\_gene\_cluster | 36 | 29.15047196 | 268 | 5.2e-71 |
| CAE14178.1 |  | BGC0001180 | Isopropylstilbene\_biosynthetic\_gene\_cluster | 34 | 29.372570794 | 268 | 6.8e-71 |
| ACY06290.1 | type\_I\_polyketide\_synthase | BGC0001042 | Sanglifehrin\_A\_biosynthetic\_gene\_cluster | 37 | 26.0410882843 | 268 | 6.8e-71 |
| ADB23402.1 | polyketide\_synthase\_type\_I | BGC0001062 | TLN-05220\_biosynthetic\_gene\_cluster | 40 | 23.98667407 | 267 | 8.8e-71 |
| CCA89328.1 | mixed\_trans-AT\_type\_I\_polyketide\_synthase/nonribosomal\_peptide\_synthetase | BGC0001111 | Rhizopodins\_biosynthetic\_gene\_cluster | 34 | 29.3170460855 | 267 | 8.8e-71 |
| AAM12913.2 | MmpD | BGC0000182 | Mupirocin\_biosynthetic\_gene\_cluster | 36 | 29.372570794 | 267 | 1.2e-70 |
| CAJ57408.1 | polyketide\_synthase\_type\_I | BGC0000176 | Difficidin\_biosynthetic\_gene\_cluster | 35 | 33.0927262632 | 267 | 1.5e-70 |
| ABI91464.1 | beta-ketoacyl\_synthase | BGC0001094 | Enacyloxin\_biosynthetic\_gene\_cluster | 34 | 30.5941143809 | 267 | 1.5e-70 |
| AIC32693.1 | FR9DEF | BGC0001113 | Spliceostatin\_/\_FR901464\_biosynthetic\_gene\_cluster | 35 | 29.8722931705 | 266 | 2e-70 |
| AIU36104.1 | LglE | BGC0000180 | Legioliulin\_biosynthetic\_gene\_cluster | 33 | 28.7062742921 | 266 | 2e-70 |
| ADH01487.1 | polyketide\_synthase | BGC0001096 | FR901464\_biosynthetic\_gene\_cluster | 35 | 29.8722931705 | 266 | 2e-70 |
| CAG23977.1 | polyketide\_synthase\_type\_I | BGC0000176 | Difficidin\_biosynthetic\_gene\_cluster | 35 | 28.0955024986 | 266 | 2.6e-70 |
| ALD83702.1 | tAT\_polyketide\_synthase | BGC0001299 | Chlorotonil\_biosynthetic\_gene\_cluster | 34 | 30.1499167129 | 266 | 2.6e-70 |
| AGN11881.1 | TstDEF | BGC0001114 | Thailanstatin\_biosynthetic\_gene\_cluster | 36 | 29.3170460855 | 265 | 3.4e-70 |
| CTQ34883.1 | AtcF;\_polyketide\_synthase,\_modules\_8-10 | BGC0001301 | Anthracimycin\_biosynthetic\_gene\_cluster | 37 | 28.4841754581 | 265 | 4.4e-70 |
| AFX60334.1 | polyketide\_synthase | BGC0001032 | Oocydin\_A\_biosynthetic\_gene\_cluster | 35 | 28.928373126 | 265 | 4.4e-70 |
| CCC21123.1 | type-I\_polyketide\_synthases | BGC0000171 | 9-methylstreptimidone\_biosynthetic\_gene\_cluster | 35 | 29.816768462 | 265 | 5.7e-70 |
| CAN89634.1 | putative\_polyketide\_synthase | BGC0001070 | Kirromycin\_biosynthetic\_gene\_cluster | 37 | 29.9833425875 | 265 | 5.7e-70 |
| CTQ34882.1 | AtcE;\_polyketide\_synthase,\_modules\_5-7 | BGC0001301 | Anthracimycin\_biosynthetic\_gene\_cluster | 36 | 26.3187118268 | 264 | 7.5e-70 |
| ACY01400.1 | AT-less\_polyketide\_synthase | BGC0000083 | Lactimidomycin\_biosynthetic\_gene\_cluster | 37 | 26.0966129928 | 264 | 7.5e-70 |
| BAP34740.1 | type\_I\_polyketide\_synthase | BGC0000078 | Incednine\_biosynthetic\_gene\_cluster | 36 | 24.1532481954 | 264 | 7.5e-70 |
| CAP95405.1 |  | BGC0001404 | Sorbicillin\_biosynthetic\_gene\_cluster | 35 | 25.8745141588 | 264 | 7.5e-70 |
| AAS47562.1 | mixed\_type\_I\_polyketide\_synthase\_-\_peptide\_synthetase | BGC0001108 | Pederin\_biosynthetic\_gene\_cluster | 34 | 30.2054414214 | 264 | 9.8e-70 |
| CCA89329.1 | trans-AT\_type\_I\_polyketide\_synthase | BGC0001111 | Rhizopodins\_biosynthetic\_gene\_cluster | 35 | 29.8722931705 | 264 | 9.8e-70 |
| ctg1\_orf8 |  | BGC0001109 | Pederin\_biosynthetic\_gene\_cluster | 34 | 30.2054414214 | 264 | 9.8e-70 |
| ABS90470.1 | NRPS/PKS | BGC0001106 | Oxazolomycin\_biosynthetic\_gene\_cluster | 35 | 27.7623542476 | 263 | 1.7e-69 |
| CAL69890.1 | RhiC\_protein | BGC0001112 | Rhizoxins\_biosynthetic\_gene\_cluster | 35 | 29.372570794 | 263 | 1.7e-69 |
| AAY89050.1 | polyketide\_synthase | BGC0001069 | Chivosazole\_biosynthetic\_gene\_cluster | 35 | 31.6490838423 | 262 | 2.8e-69 |
| AAY89053.1 | polyketide\_synthase | BGC0001069 | Chivosazole\_biosynthetic\_gene\_cluster | 36 | 27.6513048306 | 262 | 3.7e-69 |
| CAG23968.1 | polyketide\_synthase\_type\_I | BGC0000181 | Macrolactin\_biosynthetic\_gene\_cluster | 33 | 30.8717379234 | 262 | 3.7e-69 |
| ADN68478.1 | SorC | BGC0000184 | Sorangicin\_biosynthetic\_gene\_cluster | 35 | 29.483620211 | 262 | 4.9e-69 |
| ABC33986.1 | polyketide\_synthase,\_putative | BGC0000186 | Thailandamide\_biosynthetic\_gene\_cluster | 34 | 28.5397001666 | 261 | 6.3e-69 |
| CBW45749.1 | hybrid\_PKS/NRPS\_(PKSI) | BGC0000952\_c3 | Pristinamycin\_biosynthetic\_gene\_cluster | 35 | 29.705719045 | 261 | 6.3e-69 |
| ADA69237.1 | trans-AT\_polyketide\_synthase | BGC0001071 | Nosperin\_biosynthetic\_gene\_cluster | 31 | 31.3714602998 | 261 | 6.3e-69 |
| AMH40423.1 | PKS | BGC0001350 | Phormidolide\_biosynthetic\_gene\_cluster | 35 | 28.7617990006 | 261 | 6.3e-69 |
| ABI91469.1 | beta-ketoacyl\_synthase | BGC0001094 | Enacyloxin\_biosynthetic\_gene\_cluster | 35 | 28.2620766241 | 261 | 8.3e-69 |
| AIJ04680.1 | polyketide\_synthase | BGC0001383 | Macrolactin\_biosynthetic\_gene\_cluster | 32 | 28.9838978345 | 260 | 1.1e-68 |
| AFX60340.1 | polyketide\_synthase | BGC0001032 | Oocydin\_A\_biosynthetic\_gene\_cluster | 32 | 30.0943920044 | 260 | 1.1e-68 |
| AJO72742.1 | Type\_I\_modular\_polyketide\_synthase | BGC0001381 | Brasilinolide\_biosynthetic\_gene\_cluster | 36 | 26.4852859522 | 260 | 1.4e-68 |
| AIJ04686.1 | polyketide\_synthase | BGC0001383 | Macrolactin\_biosynthetic\_gene\_cluster | 33 | 30.8717379234 | 260 | 1.4e-68 |
| AEH59109.1 | keto-hydroxyglutarate-aldolase/polyketide\_synthase | BGC0000385 | Lysobactin\_biosynthetic\_gene\_cluster | 34 | 29.816768462 | 260 | 1.4e-68 |
| BAB69199.1 | modular\_polyketide\_synthase | BGC0000117 | Oligomycin\_biosynthetic\_gene\_cluster | 35 | 25.2637423654 | 260 | 1.8e-68 |
| CAG23965.1 | polyketide\_synthase\_type\_I | BGC0000181 | Macrolactin\_biosynthetic\_gene\_cluster | 32 | 28.9838978345 | 260 | 1.8e-68 |
| ABC84458.1 | NigAIII | BGC0000114 | Nigericin\_biosynthetic\_gene\_cluster | 35 | 25.2637423654 | 259 | 2.4e-68 |
| AEC04357.1 | polyketide\_synthase | BGC0000178 | Elansolid\_biosynthetic\_gene\_cluster | 33 | 29.6501943365 | 259 | 2.4e-68 |
| CCA89327.1 | trans-AT\_type\_I\_polyketide\_synthase | BGC0001111 | Rhizopodins\_biosynthetic\_gene\_cluster | 32 | 32.8151027207 | 259 | 2.4e-68 |
| AGN74892.1 | nonribosomal\_peptide\_synthetase/polyketide\_synthase\_hybrid\_protein | BGC0000459 | Griseoviridin\_/\_viridogrisein\_biosynthetic\_gene\_cluster | 36 | 29.816768462 | 259 | 3.1e-68 |
| AAO65790.1 | ketosynthase-like\_protein | BGC0000100 | Monensin\_biosynthetic\_gene\_cluster | 29 | 39.2004441977 | 258 | 5.4e-68 |
| EAU29808.1 | hypothetical\_protein | BGC0001400 | Citreoviridin\_biosynthetic\_gene\_cluster | 28 | 48.2509716824 | 258 | 5.4e-68 |
| AEZ53953.1 | polyketide\_synthase | BGC0000144 | Salinomycin\_biosynthetic\_gene\_cluster | 30 | 37.1460299833 | 258 | 7e-68 |
| AFL48521.1 | beta-ketosynthase | BGC0000084 | Laidlomycin\_biosynthetic\_gene\_cluster | 29 | 38.3120488617 | 257 | 9.1e-68 |
| BAP05589.1 | CalA | BGC0000967 | Calyculin\_biosynthetic\_gene\_cluster | 34 | 29.6501943365 | 257 | 1.2e-67 |
| AKQ22697.1 | malonyl\_CoA-acyl\_carrier\_protein\_transacylase | BGC0001186 | Misakinolide\_biosynthetic\_gene\_cluster | 30 | 30.1499167129 | 257 | 1.2e-67 |
| AFX60317.1 | polyketide\_synthase | BGC0001031 | Oocydin\_A\_biosynthetic\_gene\_cluster | 33 | 29.594669628 | 257 | 1.6e-67 |
| CAN89632.1 | putative\_polyketide\_synthase | BGC0001070 | Kirromycin\_biosynthetic\_gene\_cluster | 35 | 28.9838978345 | 256 | 2.7e-67 |
| ACY01390.1 | AT-less\_polyketide\_synthase | BGC0000177 | Dorrigocin\_/\_migrastatin\_biosynthetic\_gene\_cluster | 33 | 29.15047196 | 255 | 4.5e-67 |
| AGS06887.1 | mixed\_type\_I\_polyketide\_synthase/non-ribosomal\_peptide\_synthetase,\_PedF-like\_protein | BGC0001092\_c2 | Diaphorin\_biosynthetic\_gene\_cluster | 35 | 29.6501943365 | 255 | 5.9e-67 |
| ACY01402.1 | AT-less\_polyketide\_synthase | BGC0000083 | Lactimidomycin\_biosynthetic\_gene\_cluster | 34 | 30.7051637979 | 254 | 7.7e-67 |
| CAN89635.1 | putative\_polyketide\_synthase | BGC0001070 | Kirromycin\_biosynthetic\_gene\_cluster | 33 | 30.8717379234 | 254 | 7.7e-67 |
| AEC04356.1 | polyketide\_synthase | BGC0000178 | Elansolid\_biosynthetic\_gene\_cluster | 30 | 36.1465852304 | 254 | 1e-66 |
| ACY01392.1 | AT-less\_polyketide\_synthase | BGC0000177 | Dorrigocin\_/\_migrastatin\_biosynthetic\_gene\_cluster | 32 | 30.5941143809 | 253 | 1.3e-66 |
| ADI59534.1 | CorL | BGC0001091 | Corallopyronin\_biosynthetic\_gene\_cluster | 34 | 28.3731260411 | 253 | 1.3e-66 |
| CAN93352.1 | polyketide\_synthase | BGC0000179 | Etnangien\_biosynthetic\_gene\_cluster | 33 | 29.372570794 | 253 | 2.3e-66 |
| AMH40421.1 | PKS | BGC0001350 | Phormidolide\_biosynthetic\_gene\_cluster | 33 | 29.5391449195 | 252 | 3.8e-66 |
| AAB08104.3 | polyketide\_synthase | BGC0000155\_c3 | T-toxin\_biosynthetic\_gene\_cluster | 35 | 25.0416435314 | 252 | 5e-66 |
| BAP34734.1 | type\_I\_polyketide\_synthase | BGC0000078 | Incednine\_biosynthetic\_gene\_cluster | 35 | 24.8195446974 | 251 | 8.6e-66 |
| ADN68482.1 | SorG | BGC0000184 | Sorangicin\_biosynthetic\_gene\_cluster | 33 | 29.6501943365 | 250 | 1.1e-65 |
| ACB12550.1 | Fum1 | BGC0000063 | Fumonisin\_biosynthetic\_gene\_cluster | 33 | 24.5974458634 | 249 | 3.3e-65 |
| AFK83820.1 | PtzD | BGC0001107\_c2 | Patellazole\_biosynthetic\_gene\_cluster | 34 | 31.4269850083 | 248 | 4.2e-65 |
| ADN68477.1 | SorB | BGC0000184 | Sorangicin\_biosynthetic\_gene\_cluster | 34 | 29.039422543 | 248 | 7.2e-65 |
| ABC38101.1 | polyketide\_synthase | BGC0000964 | Burkholdac\_biosynthetic\_gene\_cluster | 34 | 28.9838978345 | 246 | 2.1e-64 |
| AGN74894.1 | nonribosomal\_peptide\_synthetase/polyketide\_synthase\_hybrid\_protein | BGC0000459 | Griseoviridin\_/\_viridogrisein\_biosynthetic\_gene\_cluster | 35 | 30.5941143809 | 246 | 2.1e-64 |
| AKQ22699.1 | malonyl\_CoA-acyl\_carrier\_protein\_transacylase | BGC0001186 | Misakinolide\_biosynthetic\_gene\_cluster | 37 | 24.6529705719 | 246 | 2.8e-64 |
| ACR12418.1 | modular\_polyketide\_synthase,\_type\_I\_PKS | BGC0000185 | Tartrolon\_biosynthetic\_gene\_cluster | 33 | 28.928373126 | 246 | 2.8e-64 |
| CAL69891.1 | RhiD\_protein | BGC0001112 | Rhizoxins\_biosynthetic\_gene\_cluster | 32 | 29.6501943365 | 245 | 4.7e-64 |
| AIC32694.1 | FR9GH | BGC0001113 | Spliceostatin\_/\_FR901464\_biosynthetic\_gene\_cluster | 34 | 29.372570794 | 245 | 4.7e-64 |
| AAD43562.2 | Fum1p | BGC0000062 | Fumonisin\_biosynthetic\_gene\_cluster | 33 | 25.3192670738 | 244 | 1e-63 |
| CBW45741.1 | hybrid\_PKS/NRPS\_(PKSI) | BGC0000952\_c3 | Pristinamycin\_biosynthetic\_gene\_cluster | 35 | 27.7068295391 | 243 | 1.4e-63 |
| CAG23959.2 | polyketide\_synthase | BGC0001089 | Bacillaene\_biosynthetic\_gene\_cluster | 33 | 28.8173237091 | 243 | 1.4e-63 |
| ANF07288.1 | hrPKS | BGC0001340 | Byssochlamic\_acid\_biosynthetic\_gene\_cluster | 34 | 24.2087729039 | 243 | 1.8e-63 |
| AGS06824.1 | mixed\_type\_I\_polyketide\_synthase/non-ribosomal\_peptide\_synthetase,\_PedH-like\_protein | BGC0001092 | Diaphorin\_biosynthetic\_gene\_cluster | 33 | 28.8728484175 | 243 | 2.3e-63 |
| AGN11882.1 | TstGH | BGC0001114 | Thailanstatin\_biosynthetic\_gene\_cluster | 34 | 29.3170460855 | 243 | 2.3e-63 |
| AAY32966.1 | DszC | BGC0001093 | Disorazole\_biosynthetic\_gene\_cluster | 34 | 30.5941143809 | 242 | 3e-63 |
| CAL69889.1 | RhiB\_protein | BGC0001112 | Rhizoxins\_biosynthetic\_gene\_cluster | 33 | 30.3164908384 | 242 | 3e-63 |
| ACR50796.1 | putative\_polyketide\_synthase | BGC0000163 | Tetronasin\_biosynthetic\_gene\_cluster | 32 | 32.2043309273 | 242 | 5.2e-63 |
| BAP05597.1 | CalI | BGC0000967 | Calyculin\_biosynthetic\_gene\_cluster | 35 | 26.6518600777 | 241 | 8.9e-63 |
| AMH40422.1 | PKS | BGC0001350 | Phormidolide\_biosynthetic\_gene\_cluster | 33 | 30.0388672959 | 240 | 1.2e-62 |
| AFR69335.1 | polyketide\_synthase\_SpiC2 | BGC0001045 | Spiruchostatin\_biosynthetic\_gene\_cluster | 33 | 29.705719045 | 240 | 1.2e-62 |
| AAY89051.1 | hybrid\_nonribosomal\_peptide\_synthetase/polyketide\_synthase | BGC0001069 | Chivosazole\_biosynthetic\_gene\_cluster | 33 | 27.9844530816 | 240 | 1.5e-62 |
| ACO79122.1 | Type\_I\_fatty\_acid\_synthase\_ArsA | BGC0000284 | Phenolic\_lipids\_biosynthetic\_gene\_cluster | 26 | 53.1926707385 | 240 | 2e-62 |
| CAN93349.1 | polyketide\_synthase | BGC0000179 | Etnangien\_biosynthetic\_gene\_cluster | 33 | 28.5397001666 | 239 | 2.6e-62 |
| AKQ22698.1 | malonyl\_CoA-acyl\_carrier\_protein\_transacylase | BGC0001186 | Misakinolide\_biosynthetic\_gene\_cluster | 31 | 29.8722931705 | 238 | 4.4e-62 |
| ALJ49911.1 | TlmG | BGC0001237 | Thiolactomycin\_biosynthetic\_gene\_cluster | 35 | 29.15047196 | 238 | 7.5e-62 |
| BAC76474.1 | type\_I\_polyketide\_synthase\_LkcC | BGC0001100 | Lankacidin\_biosynthetic\_gene\_cluster | 34 | 29.816768462 | 236 | 2.2e-61 |
| BAE93740.1 | type\_I\_polyketide\_synthase-related\_protein | BGC0000164 | Tetronomycin\_biosynthetic\_gene\_cluster | 34 | 29.8722931705 | 235 | 3.7e-61 |
| OAQ83765.1 | KR\_domain-containing\_protein | BGC0001358 | Leucinostatins\_biosynthetic\_gene\_cluster | 34 | 21.9322598556 | 235 | 4.9e-61 |
| ABC35796.1 | polyketide\_synthase,\_putative | BGC0001102 | Malleilactone\_biosynthetic\_gene\_cluster | 32 | 30.8162132149 | 234 | 8.3e-61 |
| CUI25726.1 | Polyketide\_synthase | BGC0001355 | U-68204\_biosynthetic\_gene\_cluster | 35 | 27.3736812882 | 234 | 8.3e-61 |
| AAN85522.1 | hybrid\_nonribosomal\_peptide\_synthetase\_/\_polyketide\_synthase | BGC0001101 | Leinamycin\_biosynthetic\_gene\_cluster | 33 | 31.6490838423 | 234 | 1.1e-60 |
| CUI25673.1 | Thiolactomycin\_Polyketide\_synthase | BGC0001353 | Thiolactomycin\_biosynthetic\_gene\_cluster | 36 | 30.4275402554 | 232 | 3.2e-60 |
| ALJ49922.1 | TtmG | BGC0001236 | Thiotetroamide\_biosynthetic\_gene\_cluster | 34 | 27.7068295391 | 232 | 5.4e-60 |
| AGJ76601.1 | HglE | BGC0000869 | Heterocyst\_glycolipids\_biosynthetic\_gene\_cluster | 27 | 52.359800111 | 232 | 5.4e-60 |
| CUI25743.1 | Polyketide\_synthase | BGC0001354 | U-68204\_biosynthetic\_gene\_cluster | 33 | 27.7623542476 | 231 | 7e-60 |
| ABI91468.1 | beta-ketoacyl\_synthase | BGC0001094 | Enacyloxin\_biosynthetic\_gene\_cluster | 36 | 24.8750694059 | 231 | 7e-60 |
| CUI25703.1 | Tu3010\_Polyketide\_synthase | BGC0001352 | Thiotetronate\_Tu\_3010\_biosynthetic\_gene\_cluster | 33 | 27.3181565797 | 231 | 9.2e-60 |
| CAE52339.1 | Polyketide\_non-ribosomal\_peptide\_synthase | BGC0001088 | Albicidin\_biosynthetic\_gene\_cluster | 32 | 28.5397001666 | 231 | 9.2e-60 |
| CBW45692.1 | putative\_modular\_polyketide\_synthase\_(fragment) | BGC0000952\_c3 | Pristinamycin\_biosynthetic\_gene\_cluster | 33 | 28.8728484175 | 230 | 1.2e-59 |
| AFX60341.1 | polyketide\_synthase | BGC0001032 | Oocydin\_A\_biosynthetic\_gene\_cluster | 31 | 30.2609661299 | 230 | 1.6e-59 |
| AAS47564.1 | mixed\_type\_I\_polyketide\_synthase/nonribosomal\_peptide\_synthetase | BGC0001108 | Pederin\_biosynthetic\_gene\_cluster | 31 | 32.6485285952 | 230 | 1.6e-59 |
| ctg1\_orf6 |  | BGC0001109 | Pederin\_biosynthetic\_gene\_cluster | 31 | 32.6485285952 | 230 | 1.6e-59 |
| ADO69378.1 | beta-ketoacyl\_synthase-like\_protein,\_Acyl\_transferase\_subunit | BGC0000044 | Dawenol\_biosynthetic\_gene\_cluster | 29 | 35.202665186 | 226 | 2.3e-58 |
| BAF50721.1 | hybrid\_non\_ribosomal\_peptide\_synthetase-polyketide\_synthase | BGC0001116 | Virginiamycin\_biosynthetic\_gene\_cluster | 32 | 29.483620211 | 226 | 2.9e-58 |
| ALV82446.1 | putative\_type\_I\_polyketide\_synthase | BGC0001382 | Svaricin\_biosynthetic\_gene\_cluster | 33 | 28.9838978345 | 224 | 8.6e-58 |
| AGS06823.1 | type\_I\_polyketide\_synthase,\_PedI-like\_protein | BGC0001092 | Diaphorin\_biosynthetic\_gene\_cluster | 30 | 30.4275402554 | 224 | 8.6e-58 |
| ACM79805.1 | ZmaA | BGC0001059 | Zwittermycin\_A\_biosynthetic\_gene\_cluster | 30 | 32.3153803443 | 222 | 4.3e-57 |
| ABM63529.1 | BryX | BGC0000174 | Bryostatin\_biosynthetic\_gene\_cluster | 31 | 27.2071071627 | 222 | 5.6e-57 |
| AEC04362.1 | polyketide\_synthase | BGC0000178 | Elansolid\_biosynthetic\_gene\_cluster | 32 | 27.6513048306 | 218 | 4.7e-56 |
| OAQ83760.1 | polyketide\_synthase | BGC0001358 | Leucinostatins\_biosynthetic\_gene\_cluster | 33 | 24.1532481954 | 216 | 2.3e-55 |
| BAP05590.1 | CalB | BGC0000967 | Calyculin\_biosynthetic\_gene\_cluster | 29 | 33.4258745142 | 215 | 5.2e-55 |
| AEH59110.1 | polyketide\_synthase\_of\_type\_I | BGC0000385 | Lysobactin\_biosynthetic\_gene\_cluster | 31 | 30.0388672959 | 214 | 8.9e-55 |
| AAM94794.1 | CalE8 | BGC0000033 | Calicheamicin\_biosynthetic\_gene\_cluster | 26 | 49.1393670183 | 213 | 2.6e-54 |
| ACI12948.1 | PfaA | BGC0000861 | Eicosapentaenoic\_acid\_biosynthetic\_gene\_cluster | 27 | 42.8095502499 | 212 | 4.4e-54 |
| ABM63528.1 | BryC | BGC0000174 | Bryostatin\_biosynthetic\_gene\_cluster | 28 | 29.816768462 | 211 | 9.8e-54 |
| CBJ89766.1 | Polyketide\_synthase\_involved\_in\_xenocoumacin\_synthesis | BGC0001054 | Xenocoumacin\_biosynthetic\_gene\_cluster | 35 | 25.8189894503 | 210 | 1.3e-53 |
| CAJ76289.1 | putative\_hybrid\_non-ribosomal\_peptide-polyketide\_synthetase | BGC0000972 | Colibactin\_biosynthetic\_gene\_cluster | 31 | 29.4280955025 | 210 | 1.7e-53 |
| ABC34154.1 | thiotemplate\_mechanism\_natural\_product\_synthetase | BGC0000961 | Bactobolin\_biosynthetic\_gene\_cluster | 31 | 30.0943920044 | 210 | 1.7e-53 |
| EGJ35088.1 | polyketide\_synthase\_module | BGC0001163 | 1-heptadecene\_biosynthetic\_gene\_cluster | 30 | 31.4825097168 | 209 | 3.7e-53 |
| AAL01060.1 | omega-3\_polyunsaturated\_fatty\_acid\_synthase\_PfaA | BGC0000865 | Eicoseicosapentaenoic\_acid\_biosynthetic\_gene\_cluster | 27 | 39.6446418656 | 205 | 5.4e-52 |
| AHH34189.1 | polyketide\_synthase | BGC0001162 | 1-heptadecene\_biosynthetic\_gene\_cluster | 30 | 31.4825097168 | 203 | 1.6e-51 |
| ADH01489.1 | type\_I\_polyketide\_synthase | BGC0001096 | FR901464\_biosynthetic\_gene\_cluster | 34 | 24.3753470294 | 202 | 3.5e-51 |
| CBK62729.1 |  | BGC0001115 | Thiomarinol\_biosynthetic\_gene\_cluster | 29 | 29.261521377 | 202 | 4.6e-51 |
| AME18003.1 | enediyne\_polyketie\_synthase | BGC0001378 | Tiancimycin\_biosynthetic\_gene\_cluster | 27 | 48.9172681843 | 200 | 1.7e-50 |
| ADH01484.1 | putative\_type-I\_PKS | BGC0001096 | FR901464\_biosynthetic\_gene\_cluster | 31 | 31.0938367574 | 200 | 2.3e-50 |
| AIC32692.1 | FR9C | BGC0001113 | Spliceostatin\_/\_FR901464\_biosynthetic\_gene\_cluster | 31 | 31.0938367574 | 200 | 2.3e-50 |
| BAD55609.1 | putative\_polyketide\_synthase | BGC0001027 | Nocobactin\_NA\_biosynthetic\_gene\_cluster | 32 | 24.2642976124 | 199 | 3.9e-50 |
| CCM44330.1 | putative\_polyketide-type\_polyunsaturated\_fatty\_acid\_synthase | BGC0001056 | Zeamine\_biosynthetic\_gene\_cluster | 27 | 41.8656302054 | 198 | 8.6e-50 |
| CBW45748.1 | hybrid\_PKS/NRPS\_(PKSI) | BGC0000952\_c3 | Pristinamycin\_biosynthetic\_gene\_cluster | 31 | 31.2604108828 | 196 | 1.9e-49 |
| AAM12934.1 | MmpF | BGC0000182 | Mupirocin\_biosynthetic\_gene\_cluster | 31 | 29.483620211 | 196 | 2.5e-49 |
| CAD15512.1 | probable\_polyketide\_synthase\_protein | BGC0001014 | Micacocidin\_biosynthetic\_gene\_cluster | 31 | 28.2065519156 | 195 | 4.3e-49 |
| AGN11880.1 | TstC | BGC0001114 | Thailanstatin\_biosynthetic\_gene\_cluster | 32 | 29.816768462 | 195 | 7.3e-49 |
| ABF92824.1 | polyketide\_synthase | BGC0001025 | Myxovirescin\_biosynthetic\_gene\_cluster | 29 | 28.7062742921 | 194 | 9.5e-49 |
| CAL69893.1 | RhiE\_protein | BGC0001112 | Rhizoxins\_biosynthetic\_gene\_cluster | 31 | 27.9844530816 | 193 | 2.8e-48 |
| AAB81123.1 | unknown | BGC0000862 | Eicosapentaenoic\_acid\_biosynthetic\_gene\_cluster | 26 | 39.7556912826 | 183 | 1.7e-45 |
| ACB47048.1 | DynE8 | BGC0001060 | Dynemicin\_biosynthetic\_gene\_cluster | 29 | 41.254858412 | 183 | 2.2e-45 |
| AHF22841.1 | MarX | BGC0000091 | Marineosin\_biosynthetic\_gene\_cluster | 31 | 26.0410882843 | 163 | 3.1e-39 |
| CAO98852.1 | polyketide\_synthase\_AufI | BGC0000023 | Aurafuron\_biosynthetic\_gene\_cluster | 25 | 24.5419211549 | 134 | 1.2e-30 |
| AGJ76603.1 | HglC | BGC0000869 | Heterocyst\_glycolipids\_biosynthetic\_gene\_cluster | 27 | 29.5391449195 | 133 | 2e-30 |
| CBW54671.1 | polyketide\_synthase/non\_ribosomal\_peptide\_synthetase | BGC0000971 | Cinnabaramide\_biosynthetic\_gene\_cluster | 26 | 31.9822320933 | 133 | 2.6e-30 |
| AAB81125.1 | unknown | BGC0000862 | Eicosapentaenoic\_acid\_biosynthetic\_gene\_cluster | 26 | 27.7623542476 | 124 | 1.6e-27 |
| BAC57029.1 | protomycinolide\_IV\_synthase\_2 | BGC0000102 | Mycinamicin\_biosynthetic\_gene\_cluster | 29 | 20.0999444753 | 115 | 7.3e-25 |
| AAP85362.1 | putative\_keto\_synthase\_alpha | BGC0000233 | Hedamycin\_biosynthetic\_gene\_cluster | 23 | 21.5435868962 | 77 | 1.3e-13 |
